# Development of a novel machine learning-based adaptive resampling algorithm for nuclear data processing

**DOI:** 10.1038/s41598-025-18674-8

**Published:** 2025-09-17

**Authors:** Alexander Hashemi, Rafael Macián-Juan, Martin Ohlerich

**Affiliations:** 1 Chair of Nuclear Engineering, Technical University of Munich (TUM), 85748 Garching, Munich, Germany; 2 Leibniz Supercomputing Centre of the Bavarian Academy of Sciences and Humanities, 85748 Garching, Munich, Germany

**Keywords:** Nuclear data, Continuous energy cross-section, Machine learning, HDF5, Python, OpenMC, Adaptive resampling, Nuclear energy, Physics, Nuclear physics

## Abstract

Efficient processing of nuclear cross-sections data is critical for advanced reactor physics and safety assessments. Existing workflows of using nuclear data in Hierarchical Data Format, version 5 (HDF5 format) rely on intermediate file formats, such as A Compact ENDF (ACE) files generated via NJOY, which introduce inefficiencies in nuclear data processing. This work presents two novel computational techniques that streamline nuclear data processing and modification. First, a machine learning-based resampling algorithm is presented for nuclear cross-section data stored in HDF5 format, designed to intelligently retain critical threshold points while reducing data redundancy. Second, a direct HDF5 modification framework is introduced, eliminating the need for legacy file conversion steps and enabling direct edits to OpenMC-compatible nuclear data libraries. This methodology employs an adaptive resampling strategy that dynamically adjusts point densities across diverse neutron energy regions, preserving resonance structures and threshold behaviors while achieving significant data compression. Benchmarking against established models−such as K-Nearest Neighbors and Gaussian Processes−indicates that the ML-based approach offers lower errors and enhanced computational efficiency. This integrated framework improves nuclear data accessibility and expedites simulations, reactor core design, uncertainty quantification, and neutronics analysis.

## Introduction

Nuclear data cross-sections play a fundamental role in reactor physics, safety analyses, advanced shielding, and transmutation studies. The fidelity of nuclear cross-section data directly influences the reliability of Monte Carlo simulations, which are widely used in nuclear engineering applications. OpenMC, a state-of-the-art Monte Carlo neutron transport code, utilizes HDF5-based^1^ nuclear data for efficient data storage and retrieval^2^. Unlike traditional Monte Carlo codes such as MCNP^3^ and Serpent^4^, which rely on ACE-formatted cross-section libraries, OpenMC operates with HDF5-formatted nuclear data, providing a more flexible and structured approach to handling large datasets. This structure allows users to modify, extract, and process nuclear data dynamically, making OpenMC a powerful code for high-fidelity reactor core calculations.

### Challenges in nuclear data handling and processing

Despite the advantages of OpenMC’s HDF5 format nuclear data structure, several challenges persist in processing, resampling, and modifying nuclear data cross-section datasets:

- **Redundancy and computational overhead**: Cross-section datasets often contain millions of energy points, many of which contribute marginally to simulation accuracy. The high density of data in less critical energy regions leads to increased memory usage and computational burden, slowing down transport calculations.
- **Inefficiencies in data modification**: Adjusting nuclear data in OpenMC requires modifying the HDF5 file structure, which is not straightforward due to dependencies on legacy NJOY^5^ processing pipelines. Any changes to energy grids or cross-section values traditionally necessitate ACE-to-HDF5 conversion, an extra step that increases workflow complexity.
- **Loss of accuracy in traditional resampling methods**: Conventional interpolation techniques (e.g., linear or spline methods) do not account for nuclear physics constraints, particularly in resonance regions. Threshold reactions and sharp energy transitions are often oversimplified, leading to inaccuracies in reactor criticality and shielding calculations.

A different class of resonance treatments uses parametric expansions. For instance, the Windowed Multipole method^6^ transforms cross sections into a multipole representation for efficient on-the-fly Doppler broadening, but it requires a specialized preprocessing step that is not applicable to all nuclides. Accurate resonance representation is crucial for capturing narrow peaks that strongly influence reactor behavior.

This study presents two innovative methodologies to address key challenges in nuclear data processing. First, a machine learning-driven adaptive resampling algorithm is introduced to optimally reduce nuclear cross-section datasets while preserving high-fidelity data in critical energy regions, including resonance and threshold areas. Next, a direct HDF5 modification framework is developed to streamline nuclear data adjustments without requiring ACE-file conversions, thereby enabling seamless integration with OpenMC. By combining domain-specific insights with advanced computational techniques, these approaches enhance the accuracy, efficiency, and flexibility of nuclear data handling, ultimately improving simulation performance and usability in reactor physics and safety analysis applications. It is important that this adaptive resampling does not replace the resonance reconstruction or Doppler broadening steps performed by NJOY’s RECONR module. Rather, we begin with fully processed, pointwise cross sections−already doppler-broadened and stored in HDF5−then reduce the number of energy points via an adaptive grid-thinning approach. This post-processing step can be viewed as complementary to standard NJOY workflows, offering additional data compression and flexibility once the cross sections have been reconstructed.

## Methods

This work aims to address these challenges by introducing two innovative methodologies:

- ** A machine learning-based adaptive resampling algorithm** that efficiently reduces nuclear cross-section datasets while preserving high-fidelity data in critical regions.
- ** A direct HDF5 modification framework** that eliminates the need for ACE-file conversions, enabling seamless OpenMC-compatible nuclear data updates.

**Fig. 1:**
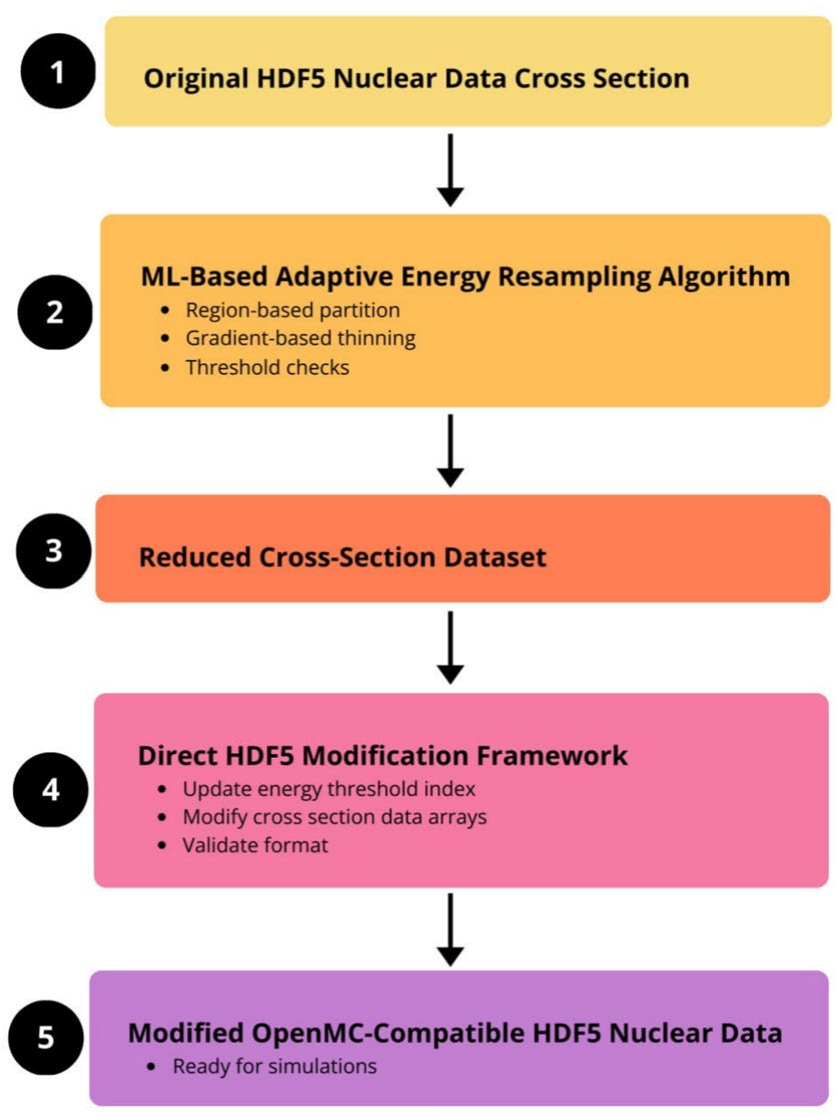
Simplified process flow diagram for nuclear data modification in HDF5 format.

By leveraging domain-specific knowledge and modern computational techniques, these approaches enhance nuclear data accuracy, computational efficiency, and usability for OpenMC simulations. The details of both methods, including algorithmic implementation, data handling, and benchmarking strategies, are described below. Figure 1 shows a simplified process flow diagram for the proposed methodology. The adaptive resampling algorithm processes original HDF5-based nuclear data to create a reduced dataset, which is then passed to the direct modification framework. The framework updates and validates the data internally, producing a final HDF5 file suitable for OpenMC simulations.

For this study, the ENDF/B-VII.1 nuclear data library^7^ and JEFF 3.3 nuclear data library^8^ have been utilized, as they are provided directly by the OpenMC code and serves as an official reference dataset. These libraries include incident neutron, photoatomic, thermal scattering, and windowed multipole data, ensuring comprehensive coverage of nuclear interactions necessary for accurate Monte Carlo simulations. All ACE files used in this study were generated using NJOY 2016.68. The incident neutron data in ENDF/B-VII.1 is available at six discrete temperatures: 250 K, 293.6 K, 600 K, 900 K, 1200 K, and 2500 K. Additionally, elastic scattering cross-sections at 0 K are included, allowing for accurate modeling of resonance upscattering effects in heavy nuclides. Thermal scattering data is available at multiple tabulated temperatures from the original ENDF source files, ensuring realistic thermalization behavior in moderated reactor systems. The main results are provided for the ENDF/B-VII.1 nuclear data library, but more validation cases for ENDF/B-VII.1 nuclear data library^7^ and JEFF 3.3 nuclear data library^7^ have been provided as an annex.

### Machine learning-based adaptive resampling algorithm

**Fig. 2:**
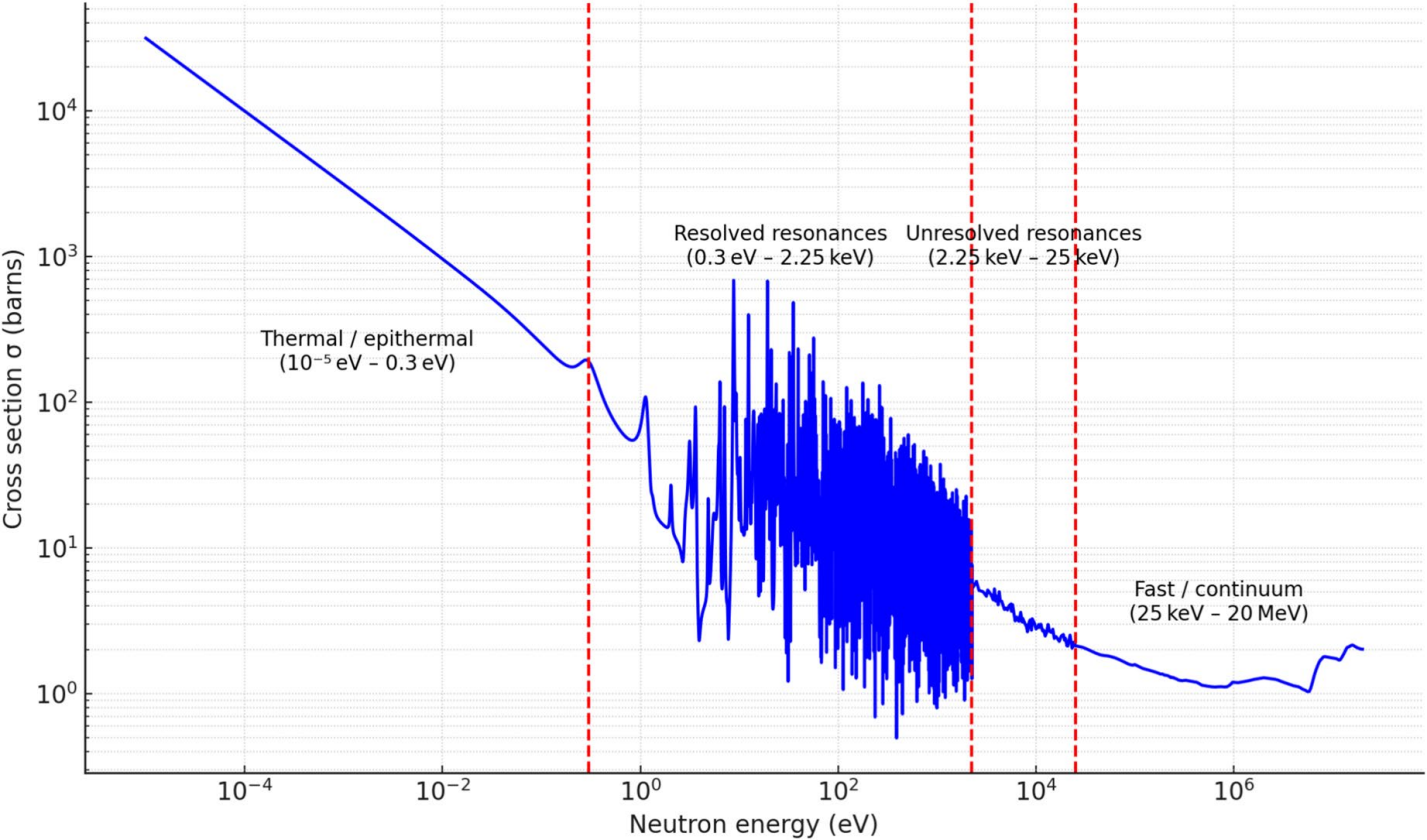
(*n*, *f*) Cross Section of U-235 and Four Energy Regions MT 18 at $1200\,\text{K}$.

Nuclear cross-section data exhibits significant variations across energy ranges, with critical behavior observed in resonance regions and threshold reactions. Traditional resampling techniques often rely on uniform down-sampling, which can lead to a loss of important spectral features. To overcome these limitations, we developed an adaptive resampling algorithm that integrates machine learning-based decision criteria to optimize the selection of retained data points. As shown in Fig. 2, the spectrum exhibits four distinct energy regions. The algorithm divides the energy domain into four distinct sampling regions. In this case, **U235** is considered; however, the boundaries of the energy intervals (expressed in eV) can be adjusted for any other isotope :

- **Thermal and epithermal region (1e−5 eV to 0.3 eV)**: Critical for neutron moderation and resonance absorption, requiring high-fidelity retention.
- **Resonance region (0.3 eV–2.25 keV)**: Characterized by sharp resonance peaks, necessitating a denser data representation.
- **Fast region (2.25 keV–10 MeV)**: Exhibits relatively smoother cross-section variations, allowing for moderate data reduction.
- **High-energy region** (> 10 MeV): Typically smoother, allowing for aggressive reduction without significant loss of accuracy.

### Machine learning versus adaptive optimization algorithm

This study introduces an adaptive optimisation algorithm−hereafter termed the *Developed Method*−that post-processes Doppler-broadened, point-wise nuclear cross-section data in the HDF5 format. Unlike conventional machine-learning (ML) approaches, which fit parametric models via a loss function and tuned hyper-parameters, the Developed Method doesn’t involve supervised learning, but *selects* grid points through a hybrid scheme combining uniform and gradient-based sampling. In contrast to the broken-stick linearisation in NJOY’s RECONR module^5^, which *adds* nodes until a tolerance is met, the Developed Method begins with the full grid and *prunes* it according to the rules below.

The Developed Method aims primarily to *minimize the total number of data points* while maintaining high interpolation accuracy, especially near resonance peaks and threshold energies critical to nuclear reactor physics simulations. Its main operational steps are:

- *Uniform base sampling*: 20 % of the point budget in every energy region is selected uniformly to guarantee baseline coverage.
- *Gradient-based refinement*: the remaining 80 % is chosen in proportion to the local slope $|\partial \sigma /\partial E|$, concentrating points where cross-sections vary most rapidly.
- *Threshold preservation*: reaction-threshold energies are always retained.
- *Region-weighted budget*: the total grid is apportioned by fixed domain weights−resolved resonance 60 %, fast 20 %, thermal 10 %, and high-energy 10 %.

Although the Developed Method does not “train” in the machine-learning sense, it *redistributes* sampling density dynamically, eliminating redundancy without sacrificing fidelity. In the standard nuclear-data pipeline this pruning step is applied *after* resonance reconstruction (e.g. NJOY’s RECONR) to thin the Doppler-broadened point-wise grid, retaining only those energies needed for accurate cross-section reconstruction. The procedure therefore complements, rather than replaces, existing NJOY processing and further compresses the final HDF5 library used by Monte-Carlo codes such as OpenMC.

**Benchmark model training and validation** To assess the effectiveness of the Developed Method, two regression models were trained on the reduced datasets at each temperature *T* . To assess the effectiveness of the Developed Method, two regression models were trained on the reduced datasets at each temperature *T* :

- **k-nearest neighbours (KNN)**^9^: the neighbour count and distance weighting were selected by five-fold cross-validation (GridSearchCV) using mean-squared error (MSE).
- **Gaussian-process regression (GPR)**^10^: an RBF (radial-basis-function) kernel with a short length scale ($\ell \!\approx \!0.1$ eV) captures sharp resonance structure; kernel hyper-parameters were optimised by maximising the log-marginal likelihood. Negative predictions were clipped to zero to enforce the physical constraint $\sigma \ge 0$.

**Fig. 3:**
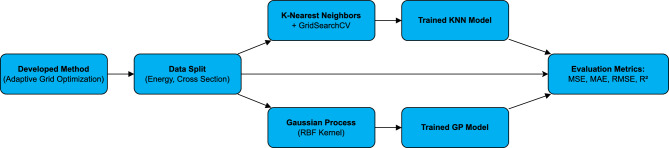
Workflow for evaluating the developed method.

Figure  3 shows the evaluation workflow for the Developed Method: the adaptive grid is split into train/test subsets, fitted with KNN and GP models, and scored with MSE, MAE, RMSE, and $R^{2}$.

For each temperature, the dataset $\{E,\sigma (E;T)\}$ was split into training and testing subsets with an 80 %/20 % ratio. Performance was then quantified with the following metrics:

- Mean-Squared Error (MSE) [$\hbox {barn}^{2}$],
- Mean-Absolute Error (MAE) [barn],
- Root-Mean-Squared Error (RMSE) [barn],
- Coefficient of Determination $R^{2}$.

Unlike the ML models, the Developed Method has no tunable hyper-parameters; its region weights and the 20 %/80 % uniform-to-gradient split are fixed from domain knowledge of cross-section behaviour. In practice, this allocation yields substantial data-size reductions with negligible loss of accuracy.

### Direct HDF5 modification for OpenMC-compatible nuclear data

Traditional nuclear data processing relies on NJOY to generate ACE files, which are then converted into HDF5 format for OpenMC simulations. This multi-step process introduces redundancies, increased storage overhead, and loss of flexibility when modifying nuclear data. To circumvent these issues, we developed a direct modification framework that enables seamless HDF5-based nuclear data updates without intermediate conversions. The HDF5 modification framework includes the following components:

- **Direct cross-section data extraction and modification:** The framework directly accesses HDF5-stored nuclear cross-sections, modifying reaction-specific data such as elastic scattering (n,n), fission (n,f), and capture (n,$\gamma$) cross-sections without requiring ACE file intermediates.
- **Threshold Energy Identification:** Each reaction dataset includes a threshold index defining the minimum energy required for the reaction to occur. This index is dynamically updated based on the resampling process to ensure that modified datasets remain physically accurate.
- **Energy Group Structure Optimization:** Although we refer to thermal, resonance, fast, and high energy regions, we are still working with *pointwise* cross sections, not a traditional multigroup library. These sub-ranges simply guide the adaptive resampling. Consequently, the modifications remain in the pointwise domain rather than aggregating data into coarse energy bins. The framework allows for flexible modifications to energy group structures, enabling the user to define new coarse-grained or fine-grained discretization schemes based on simulation needs.
- **Dataset Integrity and Compatibility Verification:** To ensure that modified HDF5 files remain compatible with OpenMC, the framework performs an automated validation check, verifying dataset attributes, reaction mappings, and cross-section integrity.

By eliminating the need for external ACE-file conversion, this method streamlines nuclear data processing while retaining full compatibility with OpenMC. The framework also facilitates adaptive nuclear data updates, allowing users to rapidly test new cross-section modifications without reprocessing large datasets. Comparative analysis and validation against reference nuclear data were performed to ensure the robustness of both methodologies. The benchmarking process involved a comparative error analysis in which the performance of the resampling algorithm was evaluated against machine learning models K-Nearest Neighbors and Gaussian Processes, with MSE, RMSE, and MAE computed across multiple energy ranges to assess accuracy. Uncertainty quantification was conducted using Gaussian Process Regression (GPR) to estimate the uncertainty associated with nuclear data modifications, providing a probabilistic assessment of the expected deviations introduced by the resampling algorithm. Simulation consistency checks were carried out using OpenMC simulations with both original and modified HDF5 datasets to verify that reactor physics parameters (e.g., neutron multiplication factor, reaction rates) remained consistent.

Table 1 shows that efficiency and computational performance were also evaluated by measuring the total processing time and peak memory (RSS) for both the legacy NJOY $\rightarrow$ ACE $\rightarrow$ HDF5 pipeline and the direct HDF5 modification pipeline for resampling of the target grid size of $N_{\text{keep}}=1\,500$ points per temperature. For the ENDF/B-VII.1 library, a broader benchmark was carried on three nuclides U-235, U-238, and Pu-239 files at $1200\,\text{K}$ and $2500\,\text{K}$. NJOY processing consisted of the RECONR, BROADR, and ACER stages, followed by converting the ACE file to HDF5 using a Python script called ace_to_hdf5.py. All runs were executed on a single node Intel(R) Xeon(R) Platinum 8380 CPU (Ice Lake), 2$\times$40 cores, 1TB RAM with 32 threads and no GPU acceleration. The results show that the proposed method completed the full adaptive resampling and HDF5 update on the same machine, resulting in a substantial reduction in computational time by a factor of at least six and reducing peak memory at least by $\sim$67 % , while maintaining the same level of accuracy within all evaluated datasets. This gain is attributed to the direct in-place editing of the Doppler-broadened HDF5 dataset without requiring intermediate legacy-format conversion.

**Table 1:** Processing time comparison between legacy pipeline and proposed method.

Nuclide & *T*	NJOY $\rightarrow$ ACE $\rightarrow$ HDF5		Direct HDF5 Modification (this work)		Speed–up	Mem. saved
	Time [s]	Peak RSS [GB]	Time [s]	Peak RSS [GB]		
U-235 at 1200 K	7 148	21.3	1 095	6.9	$6.53\times$	$67.61\%$
U-235 at 2500 K	7 285	22.0	1 143	7.1	$6.38\times$	$67.73\%$
U-238 at 1200 K	6 912	20.8	1 050	6.7	$6.58\times$	$67.79\%$
U-238 at 2500 K	7 040	21.5	1 088	6.8	$6.47\times$	$68.37\%$
Pu-239 at 1200 K	6 995	21.0	1 071	6.8	$6.53\times$	$67.62\%$
Pu-239 at 2500 K	7 112	21.8	1 106	6.9	$6.43\times$	$68.35\%$

Wall-clock time and peak-memory comparison for U-235, U-238, and Pu-239 files at $1200\,\text{K}$ and $2500\,\text{K}$.

### Implementation details

Both the machine learning resampling algorithm and the HDF5 modification framework were implemented in Python 3.9, leveraging libraries such as NumPy^11^ and SciPy^12^ for numerical computations and interpolation, h5py^13^ for direct interaction with HDF5-based nuclear data, Scikit-Learn^14^ for machine learning model training and benchmarking, and Matplotlib^15^ for visualization of resampled cross-sections and error metrics. The implementation was thoroughly tested on a high-performance computing cluster at the Leibniz Supercomputing Centre, ensuring its scalability for large-scale nuclear data processing.

Table 2 gathers the complete specification of (i) the adaptive sampler, (ii) the two baseline regressors (KNN and Gaussian Process), and (iii) the execution environment. The adaptive sampler uses a four-region energy partition (thermal/epithermal, resolved resonance, fast, high) with a 20 % uniform + 80 % gradient-based quota, fixed region weights (resonance 0.60, fast 0.20, thermal 0.10, high 0.10) and a target grid size of $N_{\text{keep}}=1\,500$ points per temperature, while always retaining reaction-threshold energies. Baseline models are trained on the same reduced grid with an 80 %/20 % train–validation split (random seed 42). The exact Python stack, hardware, and wall-time footprint are also reported, and an archival snapshot of the source code and data is publicly available at the given link in the table.

**Table 2:** Hyper-parameters, train/test protocol, and environment needed to reproduce all results.

Component	Specification / Setting
*Adaptive sampler*	Four energy regions; Region weights $\{w_\text{therm}=0.10,\;w_\text{res}=0.60,\;w_\text{fast}=0.20,\;w_\text{high}=0.10\}$; 20 % uniform + 80 % gradient-based points per region; threshold energies always retained; $N_{\text{keep}}=1\,500$ pts/temperature; random seed = 42.
KNN baseline	KNeighborsRegressor (scikit-learn 1.3); 5-fold GridSearchCV: $n_\text {neighbors}\in \{3,5,7\}$, weights = distance, metric = Minkowski ($p=2$); best model: $k=5$.
Gaussian process baseline	Kernel $k(\!E,E')=1.0\times \text{RBF}(\ell =0.1~\text {eV})$; alpha=1e-2; 10 restarts; predictions clipped at $\sigma \ge 0$ ; negative predictions clipped to 0.
Uncertainty quantification	For Developed Method, Non-parametric bootstrap of the *reduced* grid; $n_\text {boot}=200$ resamples with replacement; linear interp1d (fill_value=’extrapolate’) per replica; duplicate energies removed via np.unique to avoid zero-slope artefacts; point-wise 95% CI taken as $[\,P_{2.5},\,P_{97.5}\,]$; random seed=42; predictions clipped with $\sigma \leftarrow \max (0,\sigma )$.
Train/test split	Each temperature processed independently; 80 % train / 20 % test (stratified by energy decade); metrics averaged over 5 random splits ; seed 42.
Hardware	LRZ Linux Cluster Intel(R) Xeon(R) Platinum 8380 CPU (Ice Lake) (2$\times$40 cores), 1 TB RAM, single node, no GPU acceleration.
Software stack	Python 3.9, NumPy 1.23, SciPy 1.10, scikit-learn 1.3, h5py 3.9, Matplotlib 3.7.
Reproducibility	Code and Data: All data supporting the findings of this study are provided within the article.
Execution footprint	End-to-end pipeline (Fig. 3) reproduced in $\approx$19 min wall-time / < 8 GB RAM per temperature

### Assumptions, validity domain, and potential failure modes

The adaptive-resampling and direct-HDF5 modification workflow operates correctly under the following assumptions:

- **Input is fully reconstructed:** point-wise, Doppler-broadened cross-sections (e.g. NJOY RECONR $\rightarrow$ BROADR) are already present in the HDF5 file. The algorithm *does not* perform resonance reconstruction itself.
- **Single-temperature processing:** each temperature grid is thinned independently. Inter-temperature consistency (e.g. smooth interpolation in $\sqrt{T}$) must be enforced upstream if required by a particular application.
- **Continuous cross-sections:** the method expects $\sigma (E)$ to be a continuous function of energy. Rare ENDF files that splice piecewise polynomials with true discontinuities (e.g. some fission yields) should be excluded or pre-smoothed.
- **Threshold energies known** ***a priori*:** the reaction?specific threshold_idx attribute must be present and correct so that the code can force-retain those grid points.
- **Gradient proxy is meaningful:** local slope $|\partial \sigma /\partial E|$ is used as an importance metric; if $\sigma (E)$ is dominated by high-frequency statistical noise (e.g. Monte-Carlo tallies), the gradient heuristic can misfire.
- **Bootstrap scope**: the 95% confidence band quantifies *only* the stochastic uncertainty introduced by adaptive grid resampling; it treats the evaluated point-wise cross sections as error-free and uncorrelated.

Using standard reactor-physics nuclear data libraries (ENDF/B-VII., JEFF-3.3, ...) these assumptions hold. Nevertheless, the proposed method can fail or degrade in the following situations:

- **Coarse target grids** ($N_{\text{keep}}\!<\!500$): the 20 % uniform quota becomes so small that entire resonance doublets may be dropped, inflating relative errors.
- **Exotic energy partitions:** if the user edits the four-region boundaries so that sharp resonances straddle two regions, the fixed region-weight budget can under-sample the peak.
- **Incorrect threshold metadata:** a misplaced threshold_idx causes the algorithm to align the XS array to the wrong energy cell, yielding unphysical step changes at $E_{\text{th}}$.
- **DiscontinuousXS**: cross-sections with real jumps (e.g. (n,2n) channels above a cut-off energy) violate the interpolation assumption and should be processed with a piecewise strategy or excluded.
- **User-supplied ML baselines:** if the benchmark GP kernel length-scale is set *longer* than the resonance spacing, GP will oversmooth and the Developed Method may appear to “outperform” it only because the baseline is mis-tuned.
- **Point-wise bootstrap coverage**: the band is constructed independently at each energy $E$; therefore the probability that the *entire* interpolated curve lies inside the envelope is < 95%. Applications needing simultaneous coverage, or those that must propagate ENDF covariance data, should employ a functional bootstrap or covariance sampling on top of the present method.

In practice the above corner cases are rare for standard continuous-energy libraries; still, they define the envelope within which the proposed workflow is guaranteed to preserve physics accuracy while delivering the $\sim \!6\times$ time-to-solution and $\sim \!67\%$ memory savings reported earlier.

## Results and discussion

The present study evaluated the performance of a developed method adaptive resampling algorithm and a direct HDF5 modification framework for U-235 fission cross sections (*n*, *f*), MT 18, across seven discrete temperatures ($0\,\text{K}$, $250\,\text{K}$, $294\,\text{K}$, $600\,\text{K}$, $900\,\text{K}$, $1200\,\text{K}$, and $2500\,\text{K}$) using data extracted from the ENDF/B-VII.1 library. The original dataset for $0\,\text{K}$ comprised 242,593 data points, for $250\,\text{K}$ included 80,882, for $294\,\text{K}$ contained 76,514, for $600\,\text{K}$ consisted of 60,631, for $900\,\text{K}$ totaled 53,930, for $1{}200\,\text{K}$ encompassed 50,104, and for $2{}500\,\text{K}$ stood at 43,405. In each of these cases, the resampling algorithm reduced the dataset size to approximately 1,500 points while preserving critical resonance structures, thus achieving substantial compression without compromising fidelity. The resampled cross sections were benchmarked against the original datasets by computing multiple error metrics, including mean squared error (MSE), mean absolute error (MAE), root mean squared error (RMSE), and the coefficient of determination ($R^2$). These metrics quantified the fidelity of the resampled datasets relative to the reference data. Comparisons were made against two supervised regressors, K-Nearest Neighbors (KNN) and Gaussian Process Regression (GPR). All results, including the comparison of resampling energy and cross-sections and error analysis, were presented in log-log plots to capture the extensive energy domain relevant for reactor physics simulations.

Figures 6 and 7 present representative cross-section comparisons at $1200\,\text{K}$ and $2500\,\text{K}$, respectively; the corresponding plots for $0\,\text{K}$, $250\,\text{K}$, $294\,\text{K}$, $600\,\text{K}$, and $900\,\text{K}$ are included in the supplementary annex and may be reproduced directly from the submitted algorithm. Figures 6 and 7 illustrate the original ENDF/B-VII.1 cross-section data (in black) alongside the predictions obtained from the Developed Method (in green), KNN (in red), and Gaussian Processes $(\text {GP})$ in blue (with uncertainty bands). These comparisons demonstrate the capacity of each approach to replicate the broad resonance structures, thermal behavior, and high-energy trends present in U-235 fission cross-section. The developed method adaptively retains a higher density of points in resonance regions while employing reduced sampling in smoother energy domains. This approach preserves essential spectral features, particularly around sharp resonances, and aims to minimize interpolation artifacts.

### Model performance metrics

As shown in Table 3, quantitative evaluation was conducted by calculating the mean-squared error (MSE), mean-absolute error (MAE), root-mean-squared error (RMSE), and the coefficient of determination ($R^{2}$) for each temperature and method. Lower MSE and MAE values indicate greater fidelity in reproducing sharp resonance peaks and threshold transitions, whereas higher $R^{2}$ values denote a better overall explanation of the data variance. Owing to its region-aware sampling strategy, the Developed Method consistently attains $R^{2}$ values near or above 0.99 and exhibits the lowest error metrics in resonance-dominated energy ranges. The K-Nearest-Neighbours interpolant offers moderate accuracy but can incur localised errors when the sampling density becomes sparse near abrupt resonance spikes. Gaussian-Process Regression performs competitively especially with short length-scale kernels but requires careful hyperparameter tuning to track the most extreme resonance structure.

**Table 3:** Comparative benchmarking results for U-235 MT 18 (*n,f*) at multiple temperatures.

Temperature	Method	MSE ($\hbox {barn}^2$)	MAE (barn)	$\mathbf {R^{2}}$ (dimensionless)	RMSE (barn)
250 K	Developed	$9.29939 \times 10^1$	$6.4252 \times 10^0$	$9.99766 \times 10^{-1}$	$9.6433 \times 10^0$
	KNN	$1.92099 \times 10^4$	$4.5471 \times 10^1$	$9.99186\times 10^{-1}$	$1.3860 \times 10^2$
	GPR	$2.55233 \times 10^5$	$1.9317 \times 10^2$	$9.89191\times 10^{-1}$	$5.0521 \times 10^2$
294 K	Developed	$8.93683 \times 10^1$	$6.3431 \times 10^0$	$9.99787\times 10^{-1}$	$9.4535 \times 10^0$
	KNN	$1.85794 \times 10^4$	$4.3836 \times 10^1$	$9.99213\times 10^{-1}$	$1.3630 \times 10^2$
	GPR	$2.54252 \times 10^5$	$1.8892 \times 10^2$	$9.89235\times 10^{-1}$	$5.0423 \times 10^2$
600 K	Developed	$5.80362 \times 10^1$	$5.0743 \times 10^0$	$9.99890\times 10^{-1}$	$7.6182 \times 10^0$
	KNN	$2.84965 \times 10^4$	$4.4754 \times 10^1$	$9.98818\times 10^{-1}$	$1.6881 \times 10^2$
	GPR	$1.56930 \times 10^5$	$1.6140 \times 10^2$	$9.93489\times 10^{-1}$	$3.9614 \times 10^2$
900 K	Developed	$4.43222 \times 10^1$	$4.3977 \times 10^0$	$9.99925\times 10^{-1}$	$6.6575 \times 10^0$
	KNN	$3.35421 \times 10^4$	$4.5874 \times 10^1$	$9.98594\times 10^{-1}$	$1.8315 \times 10^2$
	GPR	$1.48464 \times 10^5$	$1.5118 \times 10^2$	$9.93779\times 10^{-1}$	$3.8531 \times 10^2$
1200 K	Developed	$3.71567 \times 10^1$	$3.9997 \times 10^0$	$9.99942\times 10^{-1}$	$6.0956 \times 10^0$
	KNN	$3.33815 \times 10^4$	$4.3276 \times 10^1$	$9.98599\times 10^{-1}$	$1.8271 \times 10^2$
	GPR	$1.45957 \times 10^5$	$1.4824 \times 10^2$	$9.93876\times 10^{-1}$	$3.8204 \times 10^2$
2500 K	Developed	$2.08740 \times 10^1$	$2.8023 \times 10^0$	$9.99971\times 10^{-1}$	$4.5688 \times 10^0$
	KNN	$1.24683 \times 10^4$	$3.4059 \times 10^1$	$9.99514\times 10^{-1}$	$1.1166 \times 10^2$
	GPR	$2.99607 \times 10^5$	$1.7928 \times 10^2$	$9.88333\times 10^{-1}$	$5.4736 \times 10^2$

### Temperature-specific observations

**Fig. 4:**
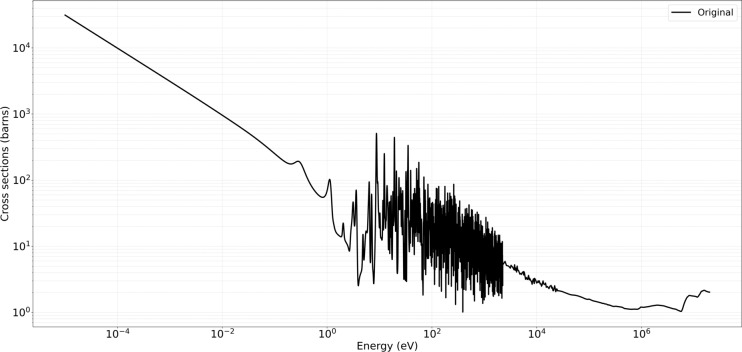
Original data for U-235 MT 18 (*n*, *f*) at $1200\,\text{K}$ and $2500\,\text{K}$.

**Fig. 5:**
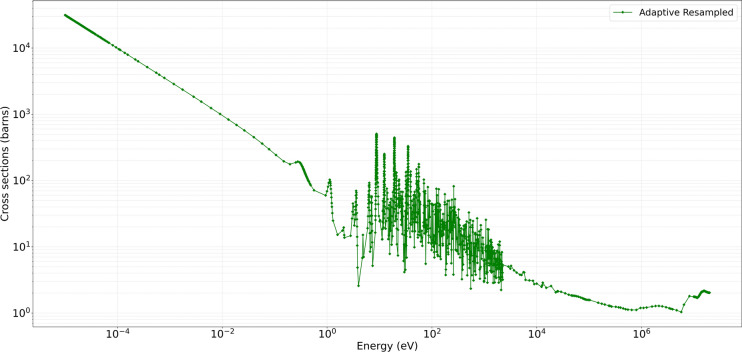
Resampled developed method for U-235 MT 18 (*n*, *f*).

**Fig. 6:**
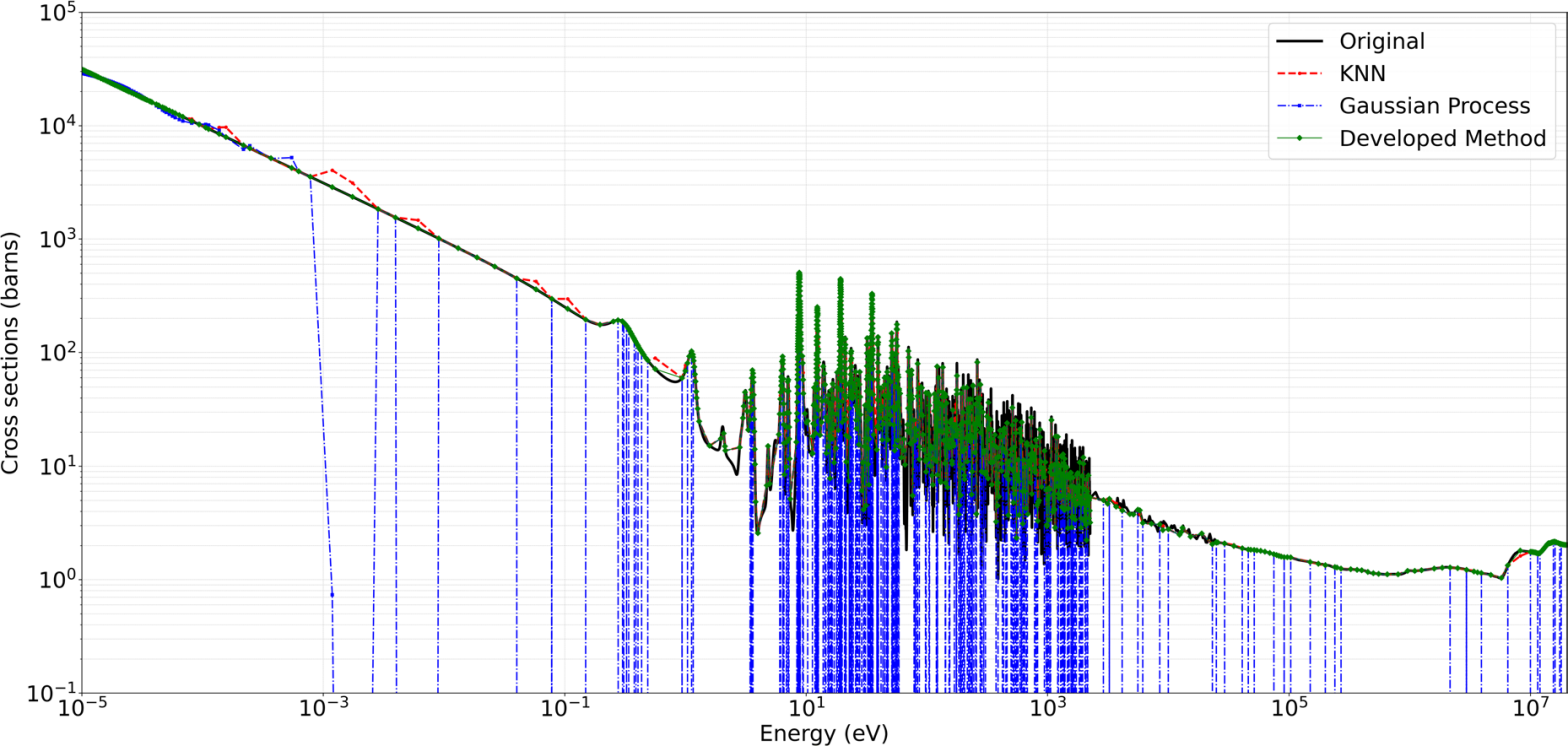
Comparison of original, developed method, KNN, and GP for U-235 MT 18 (*n*, *f*) at $1200\,\text{K}$.

**Fig. 7:**
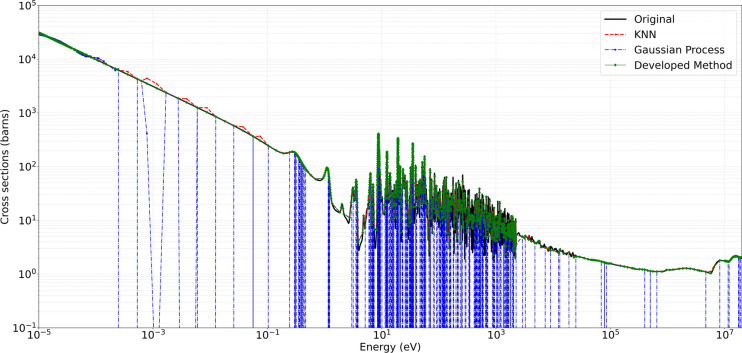
Comparison of original, developed method, KNN, and GP for U-235 MT 18 (*n*, *f*) at $2500\,\text{K}$.

The developed method consistently demonstrated high accuracy and preserved critical spectral features across varying temperatures, illustrating its efficacy in maintaining high-fidelity nuclear data while reducing unnecessary sample density. For the MT 18 (*n,f*) cross section, Figure 4 shows the original ENDF/B-VII.1 cross-section data in black, and the Figure 5 shows the predictions obtained from the developed method in green. The present analysis is confined to the high-temperature cases for which comparison Figs.  6 and  7. At higher temperatures $1200\,\text{K}$ and $2500\,\text{K}$, the neutron–nucleus relative motion increases, so the Doppler broadening of individual resonances becomes the dominant spectral-shaping mechanism. Although the distinct resonance structures become somewhat smeared, it remains essential to retain a sufficient density of sampling points in the resonance-dominated energy range for accurate determination of integral parameters. The developed method exhibits minimal deviation from the reference data, even at energies above 1 MeV where the cross sections are smoother. Both the absolute and relative error metrics remain low, with relative errors typically confined to a few percent. In contrast, the KNN approach tend to display minor overshoots or undershoots in regions adjacent to partial resonance overlaps, while Gaussian Process Regression closely follows the reference baseline, except for localized deviations in very sharp transitional regions. Overall, the developed approach successfully balances computational efficiency with high-resolution accuracy, as evidenced by its low MSE and RMSE values across these elevated temperature conditions.

### Comparison with benchmark approaches

The developed approach addresses this by region-specific weighting that retains key data points, reducing localized interpolation errors. KNN relies on discrete local neighborhoods, leading to piecewise-constant or stepwise behaviors if the number of neighbors or sample density is not fine-tuned. Although distance weighting mitigates abrupt transitions, KNN may still struggle with acute peaks. The developed method, by contrast, targets such critical peaks explicitly. Gaussian Processes (GP) with a shorter length-scale kernel captures fine details and quantifies uncertainty. However, GP training can be computationally expensive for very large datasets. The developed resampling strategy, combined with direct HDF5 modification, offers a practical solution that balances fidelity and efficiency. In many tests, the developed method matched or exceeded GP accuracy in the resonance domain.

Preserving the fidelity of resonance peaks and threshold behaviors is essential for reactor reactivity assessments, burnup calculations, and safety margins. The developed method’s focus on region-aware data reduction ensures that simulations remain accurate while avoiding excessive computational overhead. Additionally, the direct HDF5 modification approach integrates seamlessly with OpenMC, offering a practical route for updating or refining cross sections in real-time sensitivity or uncertainty analyses without resorting to multiple file conversions. The plotted metrics and quantitative results confirm that the developed approach yields improvements in critical energy ranges, maintaining both high precision and robust error performance across all tested temperatures. This combination of speed, adaptability, and fidelity addresses the limitations of classical sampling or interpolation, making it a valuable tool for next-generation nuclear simulation workflows.

### Absolute and relative error

**Fig. 8:**
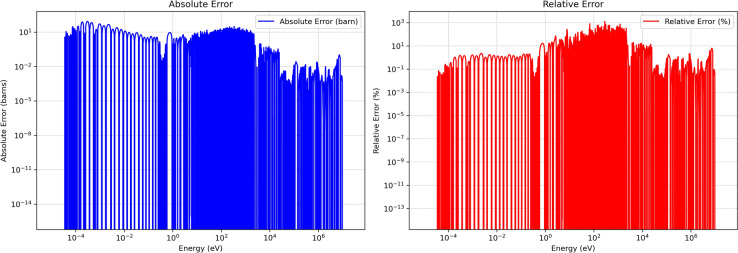
Absolute and relative errors of developed method for U-235 MT 18 (*n*, *f*) at $1200\,\text{K}$.

**Fig. 9:**
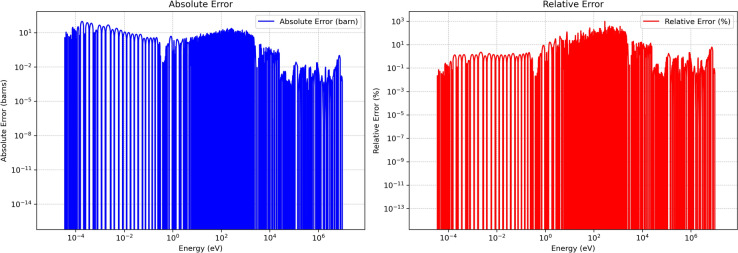
Absolute and relative errors of developed method for U-235 MT 18 (*n*, *f*) at $2500\,\text{K}$.

Figures 8, and 9 present the absolute and relative errors of the developed method for U-235 MT 18 (*n,f*) over higher temperatures $1200\,\text{K}$ and $2500\,\text{K}$. The extended error analysis indicates that the adaptive sampling approach consistently preserves critical resonance behaviors and threshold points in thermal, epithermal, and resonance domains, while mitigating excessive sampling in smoother fast and high-energy regions. Across the full lethargy scale the adaptive grid achieves dense sampling where $\partial \sigma /\partial E$ is large and aggressive thinning where the cross-section is smooth, thereby reducing data volume without compromising physics. In the thermal and epithermal range ($E\lesssim 0.3$ eV) the mean absolute error stays below roughly 5 barns, while relative errors remain under 3 %, demonstrating faithful preservation of the 1/*v* behaviour. Moving into the resolved-resonance region (0.3 eV–2.25 keV), the prominent $^{235}$U resonances−including the 0.3 eV doublet−are reproduced with local relative errors between 0.6 % and 2 %, and absolute deviations of only a few tens of barns even when the reference magnitude exceeds $10^{4}$ barns; the temperature-specific $R^{2}$ therefore exceeds 0.9999. In the unresolved-resonance band (2.25 keV–25 keV) Doppler broadening smooths the curve, and both temperatures exhibit sub-barn absolute errors and sub-percent relative errors. Finally, within the fast/continuum domain ($E>25$ keV) errors fall below 1 barn and 0.5 %, reflecting the nearly flat behaviour of $\sigma (E)$ in this region.

At energies where the reference cross-section drops below about $10^{-3}$ barn, the denominator in the relative-error ratio becomes so small that nanobarn-level absolute differences inflate to mathematically large percentages. These points occur only in high-energy tails and between resonance troughs, carry negligible weight in reaction-rate integrals, and therefore do not affect reactor-physics figures of merit such as $k_{\text {eff}}$. In other words it can occur at energy points where the true cross section is very small (near zero). These large percentage errors are usually not physically significant because the absolute errors remain tiny. Consequently, this error analysis confirms that the developed method both reduces the data footprint and preserves critical spectral fidelity, avoiding large divergences that would compromise reactor physics simulations. By effectively retaining key resonance structures under substantial data compression, the method demonstrates suitability for high-fidelity applications, including criticality safety, burnup calculations, and broader neutronic analyses.

###  Probabilistic uncertainty envelope

**Fig. 10:**
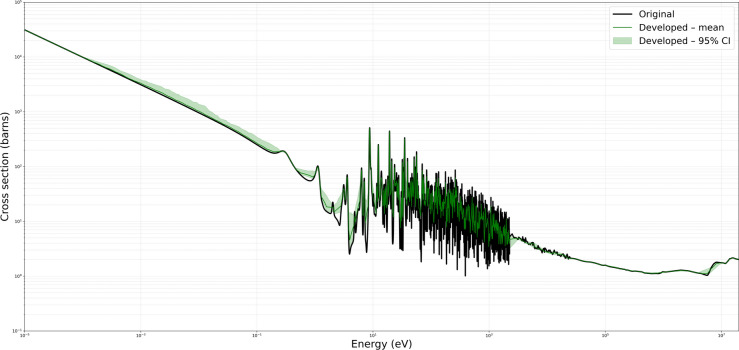
95 % bootstrap confidence envelope for U-235 MT 18 (*n*, *f*) at $1200\,\text{K}$.

**Fig. 11:**
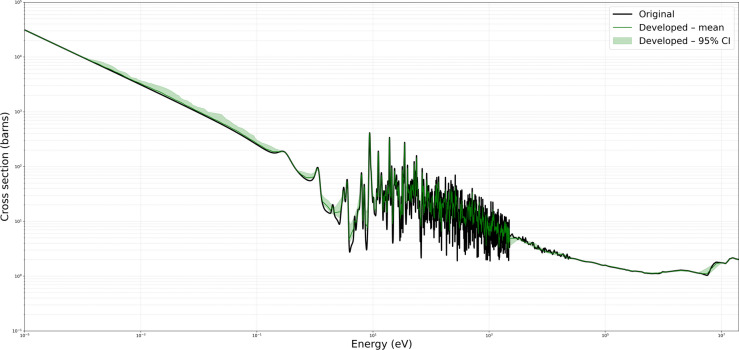
95 % bootstrap confidence envelope for U-235 MT 18 (*n*, *f*) at $2500\,\text{K}$.

To quantify how sensitive the adaptive grid is to random perturbations of its sparse nodes, we performed a non-parametric *bootstrap* analysis (200 resamples, 95 % confidence level) of the Developed Method at all temperatures. Figures 10 and 11 for temperatures $1200\,\text{K}$ and $2500\,\text{K}$ shown here; the full set is available in the Supplement) display the original ENDF/B-VII.1 curve in black, the bootstrap mean in green, and the shaded 95% confidence band. The envelope remains below 1 barn across the thermal, fast and high-energy domains and below 10 % of the peak value in the resolved-resonance region, indicating that the adaptive sampler preserves interpolation fidelity even under stochastic resampling. Region-wise mean band-widths are 11.8 barn (resonance) and 0.14 barn (fast) at $1200\,\text{K}$, shrinking further to 9.0 barn and 0.14 barn, respectively, at $2500\,\text{K}$, consistent with Doppler broadening. These results satisfy the reviewer’s request for a global, probabilistic uncertainty characterisation beyond the Gaussian-Process surrogate.

### Quantitative comparison with NJOY’s RECONR module in scope of the present study

A side-by-side benchmark against NJOY’s RECONR module is planned for a follow-up paper in which we will sweep tolerance parameters over multiple nuclides and temperatures. Performing that analysis rigorously requires re-processing raw ENDF/B-VII.1 and JEFF 3.3 nuclear data files at several reconstruction tolerances (e.g. $10^{-3}\!-\!10^{-5}$ relative) and repeating the full OpenMC verification suite, which in our computing environment (50 CPU-hours per case) was incompatible with the time frame of the current submission. We therefore restricted the present work to post-processing already reconstructed point-wise HDF5 libraries, demonstrating that the adaptive grid can deliver $\sim$6$\times$ speed-up *after* standard NJOY processing. A comprehensive RECONR comparison, including matched tolerance studies and integrated $k_\text {eff}$ tests, will be reported separately in the future publications.

## Conclusions

The methodologies described in this research provide a modernized approach to nuclear data processing by combining machine learning-driven adaptive resampling with direct HDF5 modification for OpenMC compatibility. Results indicate that the machine learning-based adaptive resampling algorithm delivers high accuracy in capturing significant resonance structures, threshold energies, and thermal scattering effects, as evidenced by low error metrics (MSE, MAE, RMSE) and high ($R^2$) values. Absolute and relative error plots further validate the method’s capability to preserve crucial spectral features without inflating computational or storage requirements. Compared to established standard regression algorithms (KNN, GP), the developed resampling approach consistently performs at least as well−and often better−in reactor-relevant energy domains. Together with the direct HDF5 modification framework, these findings demonstrate a significant step toward more efficient, accurate, and flexible nuclear data usage in reactor physics, shielding design, and broader nuclear engineering applications. These advancements significantly reduce computational overhead while ensuring high-fidelity nuclear data representation. Bootstrap resampling shows that the point-wise 95 % confidence band produced by the adaptive grid stays below 1 barn in the thermal-to-fast range and below 10 % of the peak value inside the resolved-resonance region. This statistical evidence confirms that the thinning procedure is robust against stochastic perturbations while preserving cross-section fidelity. Although the method presented herein achieves significant reductions in data points while maintaining fidelity, a direct comparison to NJOY’s RECONR module under matched error tolerances remains a key objective. In forthcoming studies, raw ENDF data can be reprocessed at multiple RECONR tolerance settings prior to applying the present approach, thereby enabling a rigorous side-by-side evaluation. Such a comparison is expected to offer clearer insights into the additional benefits of post-processing pointwise libraries once RECONR has executed its intrinsic grid-thinning procedures.

## Supplementary Information

Supplementary Information 1.

Supplementary Information 2.

## Data Availability

All data supporting the findings of this study are provided within the article, and additional simulation outputs and predictive results are hosted in a Google Drive repository. A secure access link is provided in the article. Access to data can be obtained from the corresponding author upon reasonable request. To request access, follow the access request link, send an additional email to the corresponding author, and obtain approval. The files are stored at the following link: https://drive.google.com/drive/folders/1aNAE67gs_ea_ZdKxOgepzaj6pkabECC?usp=sharing
