# Supplementary material for: Development of a novel machine learning-based adaptive resampling algorithm for nuclear data processing
Source: Sci Rep. 2025 Sep 17;15:32573. doi: 10.1038/s41598-025-18674-8 (PMC12443974; doi:10.1038/s41598-025-18674-8)
Supplement: Supplementary file 1 — Supplementary Information 1. [file 41598_2025_18674_MOESM1_ESM.pdf]

Supplementary Note
Expected Error-Budget Relationship Between NJOY's RECONR Tolerance and Adaptive Resampling

This note derives an order-of-magnitude relationship between the reconstruction tolerance used by NJOY's `RECONR` module and the residual error after adaptive resampling.

1. Two consecutive grid-control steps. NJOY's `RECONR` reconstructs resolved-resonance cross sections by *adding* points until the **relative** linear-interpolation error at each segment midpoint drops below a user-prescribed tolerance $\varepsilon_{\mathrm{rec}}$ (default 10^{-3}, or 0.1 %)}[1]. Our workflow then *removes* points from that fully reconstructed grid, guided by a physics-informed importance metric, and is designed so that the residual post-processing error $\varepsilon_{\mathrm{post}}$ satisfies

$$\varepsilon_{\mathrm{post}} \ll \varepsilon_{\mathrm{rec}}. \tag{1}$$

Because the two steps act in series, inequality (1) guarantees that the total interpolation error never exceeds the bound already certified by `RECONR`. Any speed-up obtained by adaptive thinning is therefore incremental to the accuracy secured by NJOY.

2. How many points does RECONR insert? — back-of-the-envelope. For a representative ^{235}U resolved resonance at $E_0 = 20$ eV the ENDF/B-VII.1 file lists a total width $\Gamma = 2.0 \times 10^{-2}$ eV [2]. Approximating the peak by a Lorentzian,

$$\sigma(E) = \sigma_0 \frac{\left(\frac{\Gamma}{2}\right)^2}{(E-E_0)^2 + \left(\frac{\Gamma}{2}\right)^2},$$

the standard linear-interpolation error bound for twice-differentiable functions [3] gives, for a segment half-width ΔE,

$$\frac{\delta\sigma}{\sigma} \approx \frac{(\Delta E)^2}{8\left(\frac{\Gamma}{2}\right)^2}.$$

Setting $\delta\sigma/\sigma = \varepsilon_{\mathrm{rec}}$ gives a segment spacing

$$\Delta E \le \sqrt{2\,\varepsilon_{\mathrm{rec}}}\,\Gamma \approx 0.045\,\Gamma \quad (\text{for } \varepsilon_{\mathrm{rec}} = 10^{-3}).$$

Because the full-width at half-maximum is FWHM $\approx \Gamma$, the number of segments spanning one resonance is

$$N_{\mathrm{rec}} \approx \frac{\Gamma}{\Delta E} \gtrsim 22.$$

Empirically, `RECONR` places additional nodes in the resonance *tails*, so practical grids contain $\mathscr{O}(10^2)$ points per resonance doublet.

3. Points kept by the adaptive scheme. Our post-processor accepts a target of $N_{\mathrm{keep}} \approx \mathscr{O}(10^1)$ points per resonance—obtained by the hybrid *uniform (20%)+gradient (80%)* allocation described in §Methods of the main text—while maintaining $\varepsilon_{\mathrm{post}} \lesssim 10^{-4}$. Thus the adaptive step removes roughly 90–95 % of the nodes that `RECONR` inserted, yet remains an order of magnitude below the original tolerance budget.

4. Why we defer a full numeric sweep. Performing a statistically meaningful `RECONR` comparison would require (i) regenerating point-wise libraries at several $\varepsilon_{\mathrm{rec}}$ values, (ii) repeating the full OpenMC verification suite for each case, and (iii) propagating differences to reactor metrics such as k_{eff}. On our cluster the workflow exceeds 50 CPU-hours per nuclide–temperature pair and was therefore beyond the scope of the current study. **Quantitative Comparison With NJOY's RECONR Module in Scope of the Present Study** at the end of Section **Results and Discussion** of the manuscript now clarifies that a complete tolerance sweep is planned for a follow-up publication.

References

[1] MacFarlane, R. E. & Muir, D. W. The NJOY Nuclear Data Processing System, Version 2016 (LA-UR-17-20093, 2017).

[2] Chadwick, M. B. et al. ENDF/B-VII.1 Nuclear Data Library. Nuclear Data Sheets, 112, 2887–2996 (2011).

[3] Atkinson, K. E. An Introduction to Numerical Analysis. 2nded. Wiley, New York (1989).
