# Supplementary material for: Development of a novel machine learning-based adaptive resampling algorithm for nuclear data processing
Source: Sci Rep. 2025 Sep 17;15:32573. doi: 10.1038/s41598-025-18674-8 (PMC12443974; doi:10.1038/s41598-025-18674-8)
Supplement: Supplementary file 2 — Supplementary Information 2. [file 41598_2025_18674_MOESM2_ESM.pdf]

Annex: Validation Cases from ENDF/B-VII.1 and JEFF 3.3 Nuclear Data Libraries

Here extends the evidence base by re-running the adaptive-resampling machine learning and direct-HDF5 workflow on a broader set of isotopes, temperatures, reaction channels, and nuclear-data libraries. These cases demonstrate that the proposed procedure remains accurate across evaluations, reaction mechanisms, and thermodynamic conditions. Below summarises every new test problem so that readers can quickly locate the corresponding figures. This appendix presents:

Readers can thus verify that the adaptive resampling and direct-HDF5 workflow generalises across isotopes, reactions, and major international evaluations.

- **ENDF/B-VII.1**
  - U-233 : (n, f) — MT 18 — 1200 K
  - Pu-239 : (n, f) — MT 18 — 2500 K
  - U-235 : Elastic scattering — MT 2 — 250 K
  - U-238 : Radiative capture — MT 102 — 600 K
- **JEFF-3.3**
  - U-233 : (n, f) — MT 18 — 1200 K
  - U-235 : (n, f) — MT 18 — 1200 K
  - Pu-239 : (n, f) — MT 18 — 2500 K

Collectively, these new cases demonstrate that the adaptive-resampling / direct-HDF5 pipeline preserves its accuracy across isotopes, reaction channels, and the leading international nuclear-data evaluations.

U-233 from ENDF/B-VII.1 Nuclear Data Library

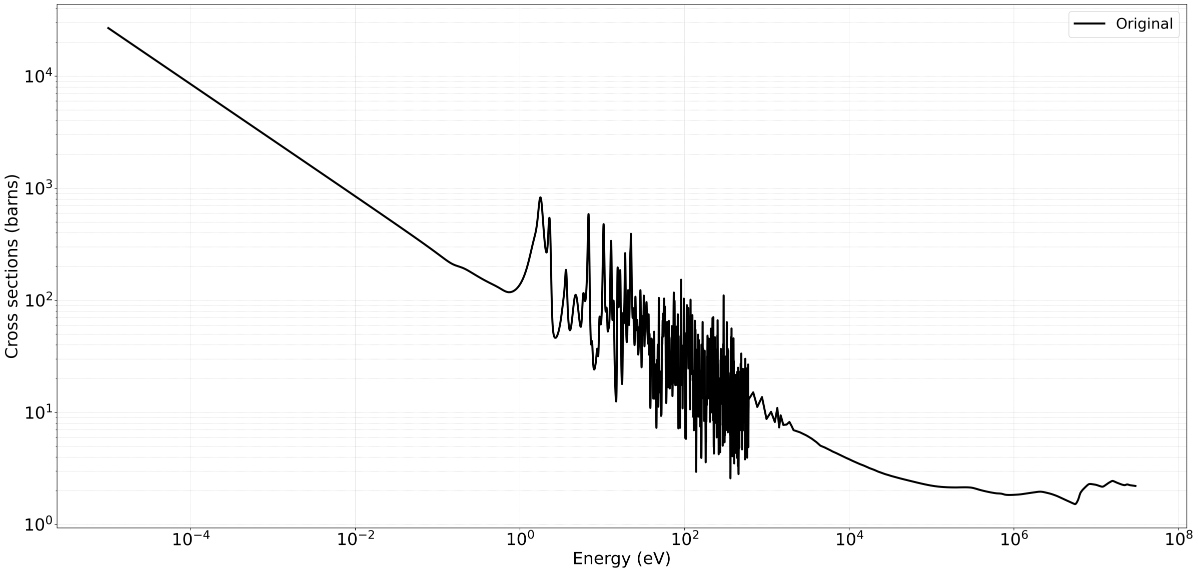

Figure 1. Original Data for U-233 MT 18 (n, f) at 1200 K in ENDF/B-VII.1

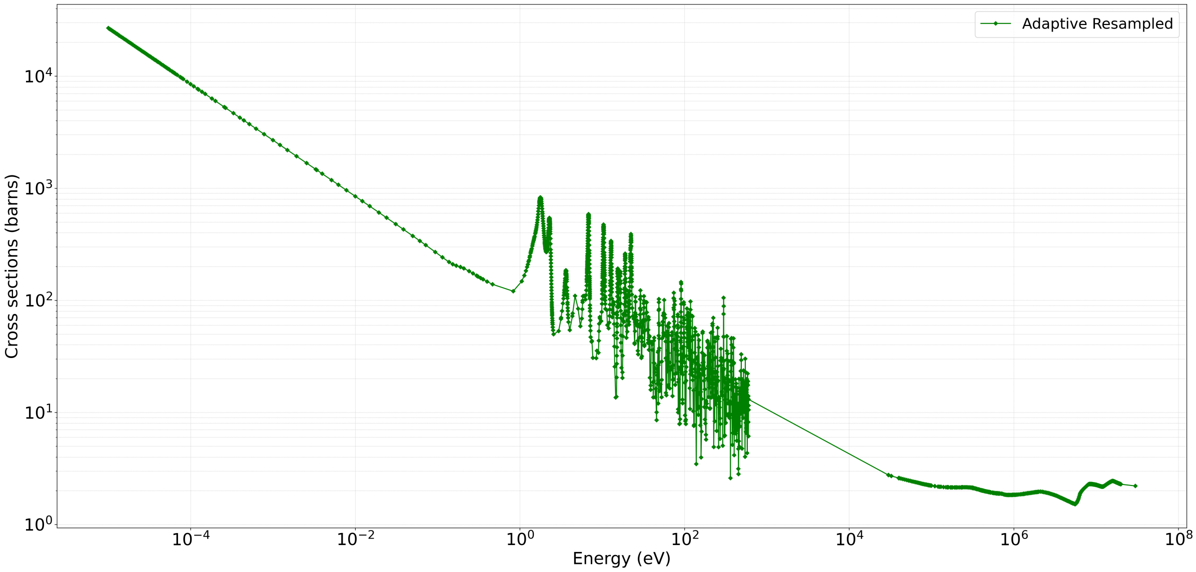

Figure 2. Resampled Developed Method for U-233 MT 18 (n, f) at 1200 K in ENDF/B-VII.1

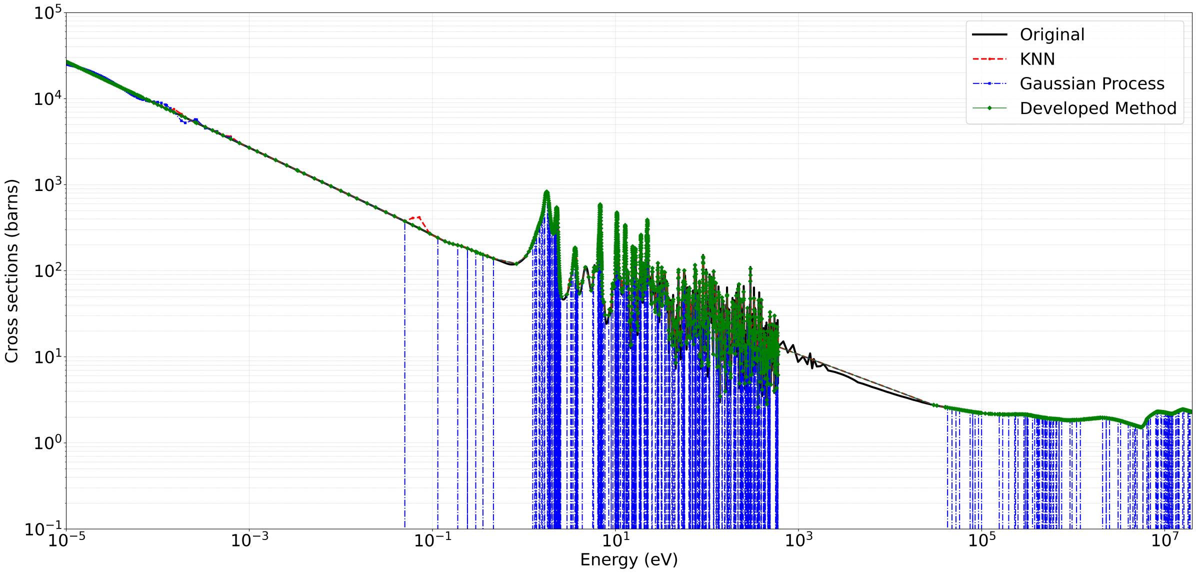

Figure 3. Comparison of Original, Developed Method, KNN, and GP for U-233 MT 18 (n, f) at 1200 K in ENDF/B-VII.1

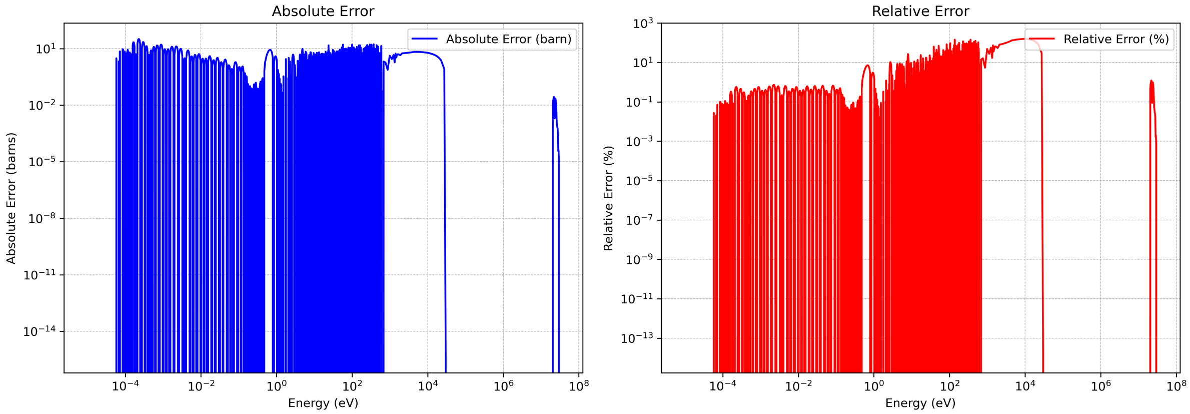

Figure 4. Absolute and Relative Errors of Developed Method for U-233 MT 18 (n, f) at 1200 K in ENDF/B-VII.1

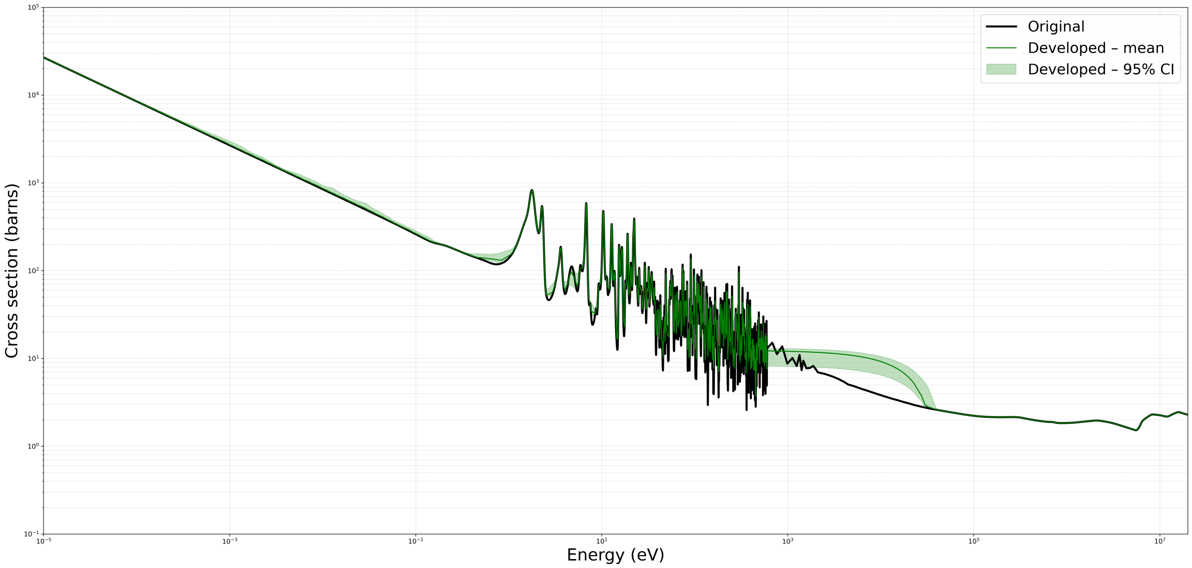

Figure 5. 95 % bootstrap confidence envelope for U-233 MT 18 (n, f) at 1200 K in ENDF/B-VII.1

U-233 from JEFF 3.3 Nuclear Data Library

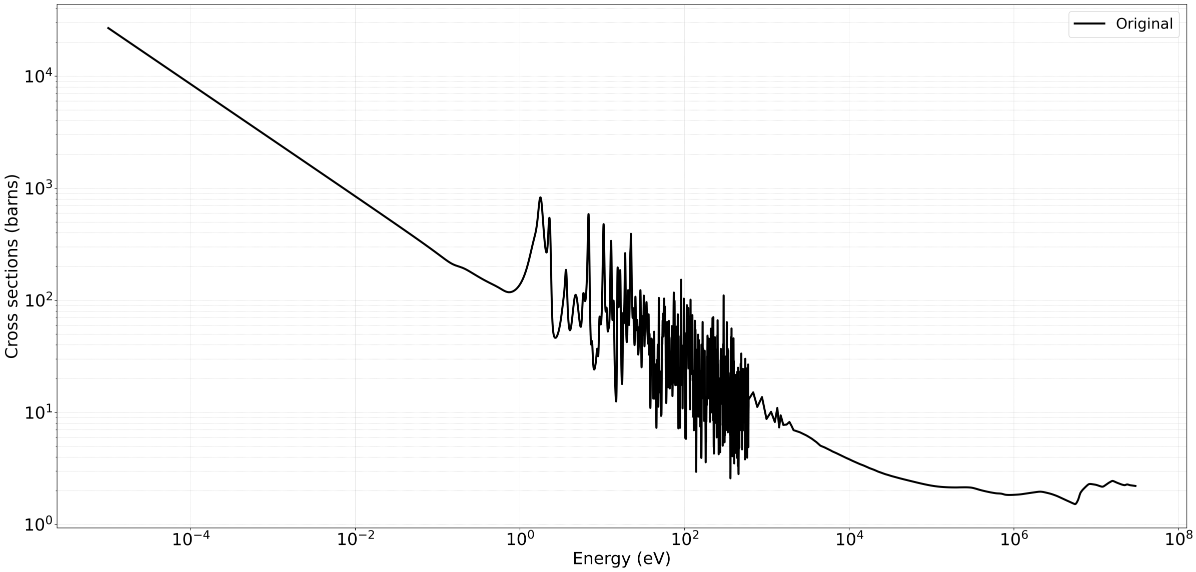

Figure 6. Original Data for U-233 MT 18 (n, f) at 1200 K in JEFF 3.3

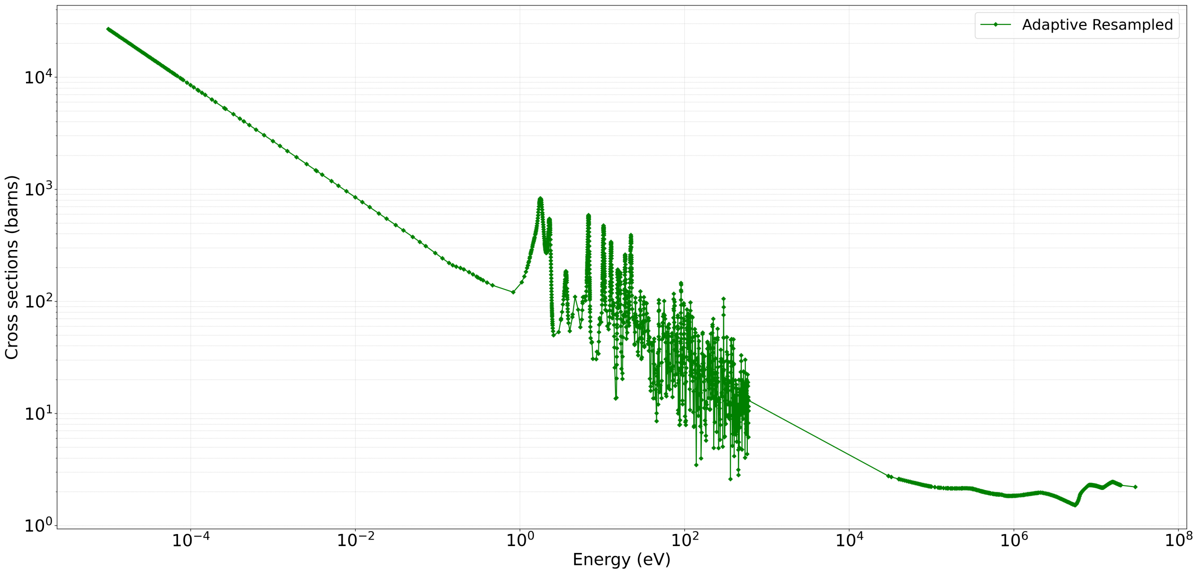

Figure 7. Resampled Developed Method for U-233 MT 18 (n, f) at 1200 K in JEFF 3.3

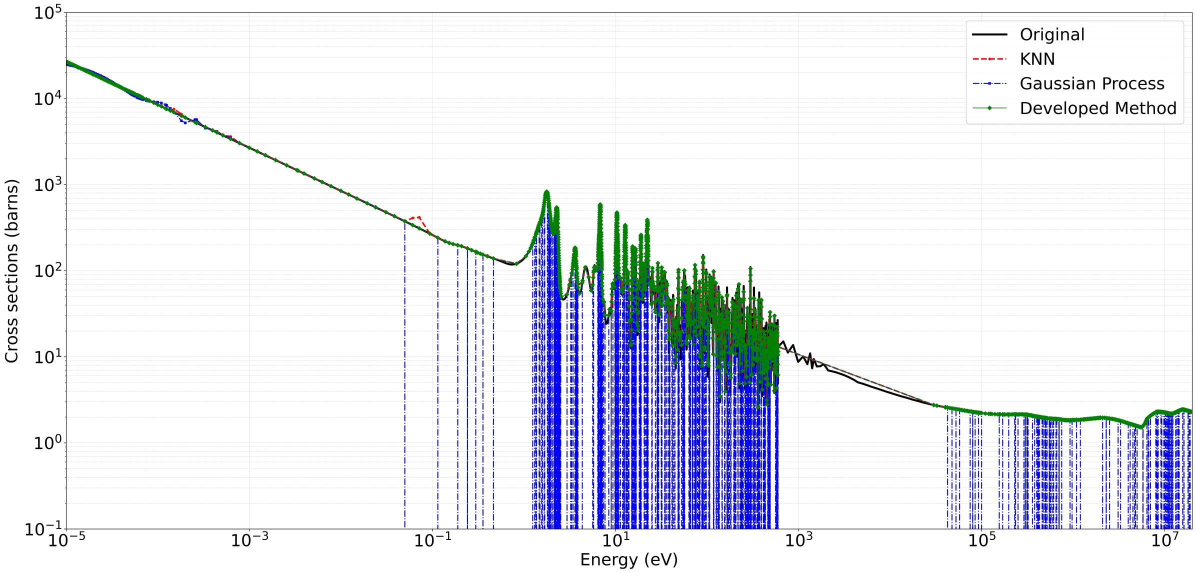

Figure 8. Comparison of Original, Developed Method, KNN, and GP for U-233 MT 18 (n, f) at 1200 K in JEFF 3.3

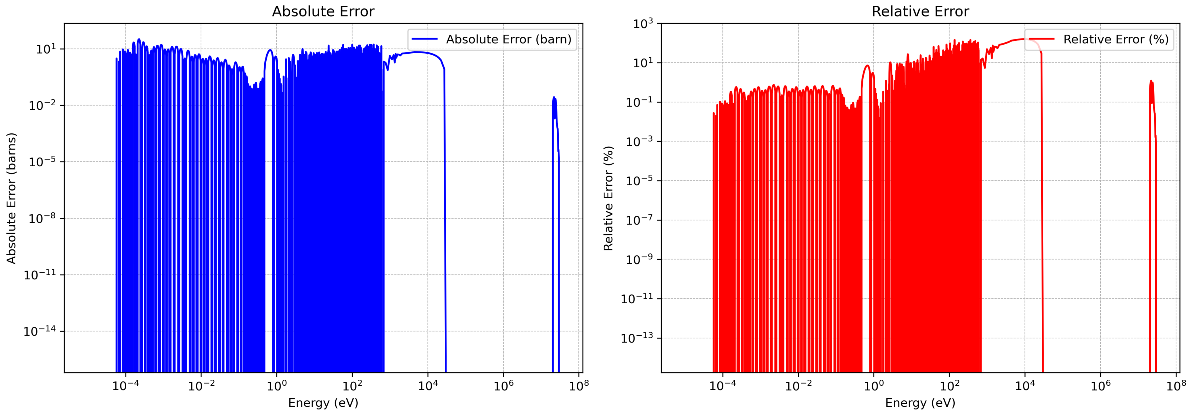

Figure 9. Absolute and Relative Errors of Developed Method for U-233 MT 18 (n, f) at 1200 K in JEFF 3.3

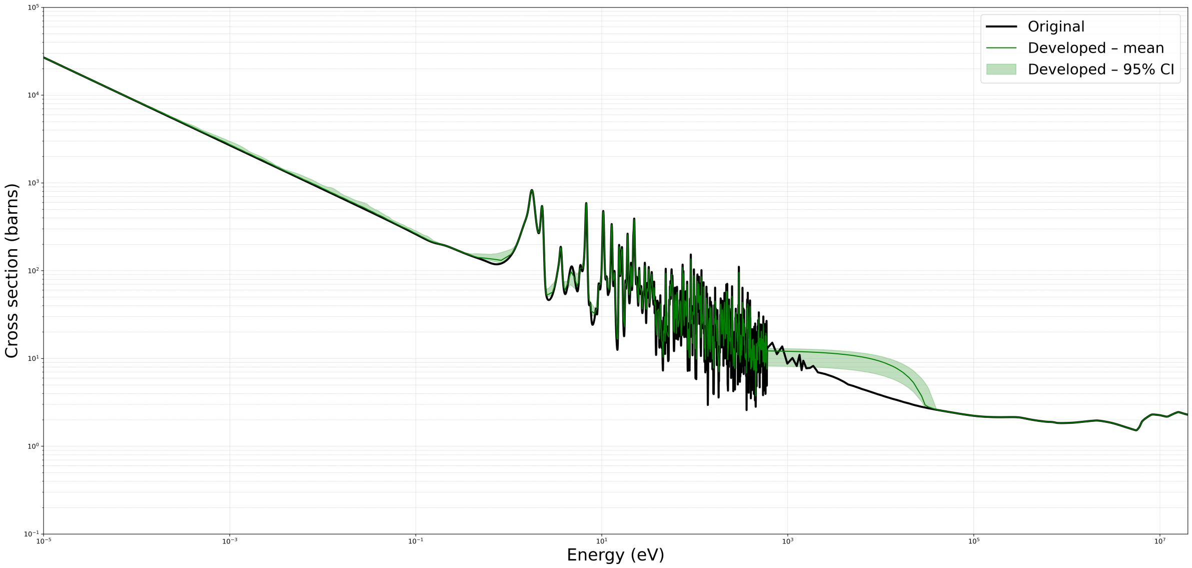

Figure 10. 95 % bootstrap confidence envelope for U-233 MT 18 (n, f) at 1200 K in JEFF 3.3

Pu-239 from ENDF/B-VII.1 Nuclear Data Library

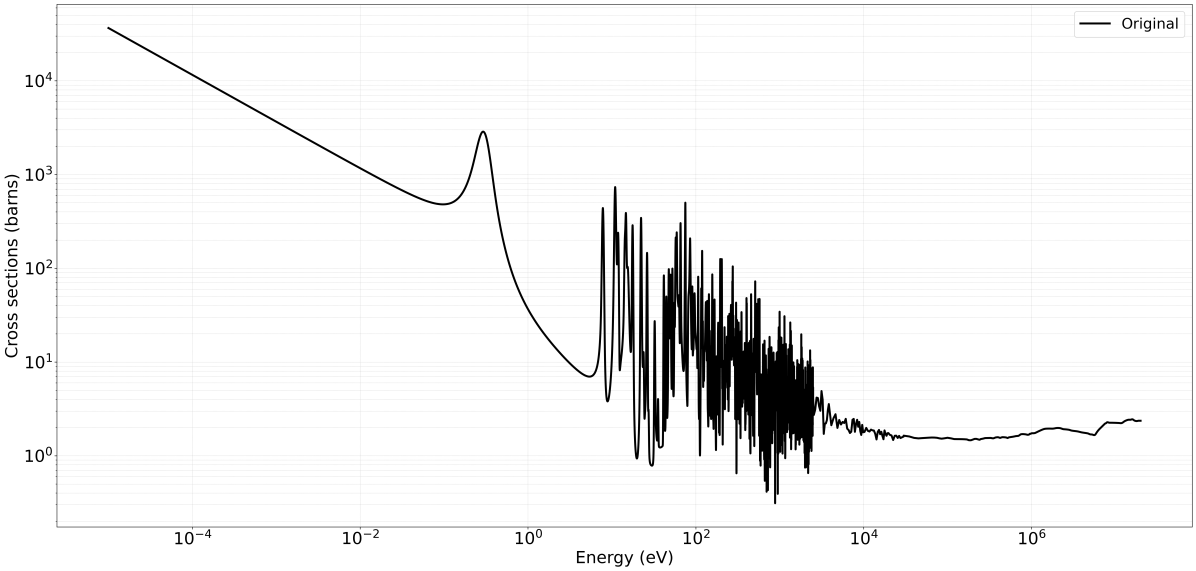

Figure 11. Original Data for Pu-239 MT 18 (n, f) at 2500 K in ENDF/B-VII.1

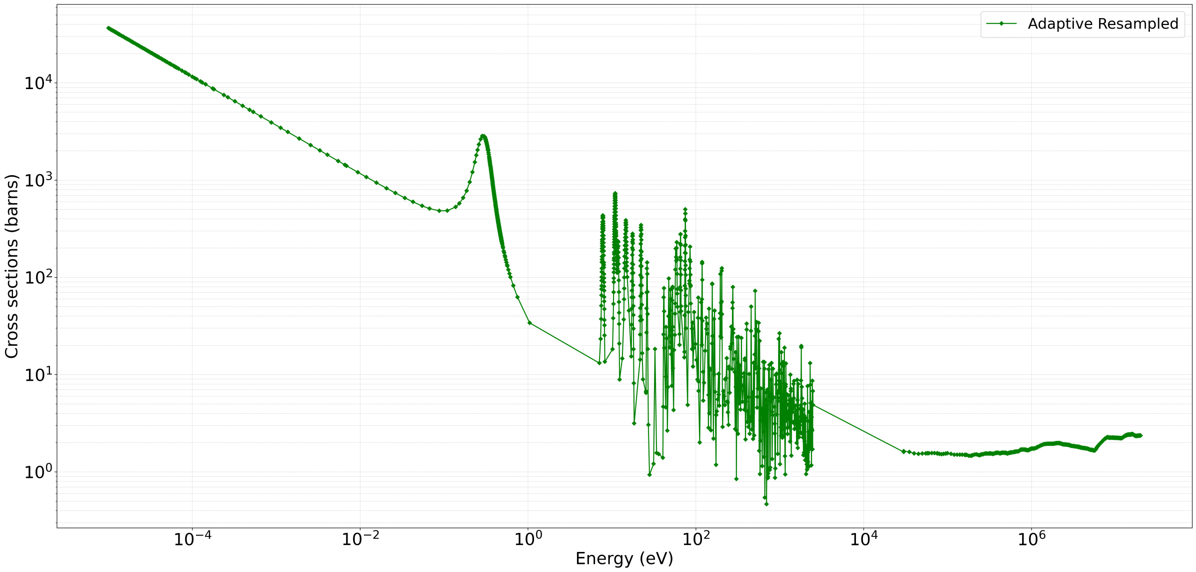

Figure 12. Resampled Developed Method for Pu-239 MT 18 (n, f) at 2500 K in ENDF/B-VII.1

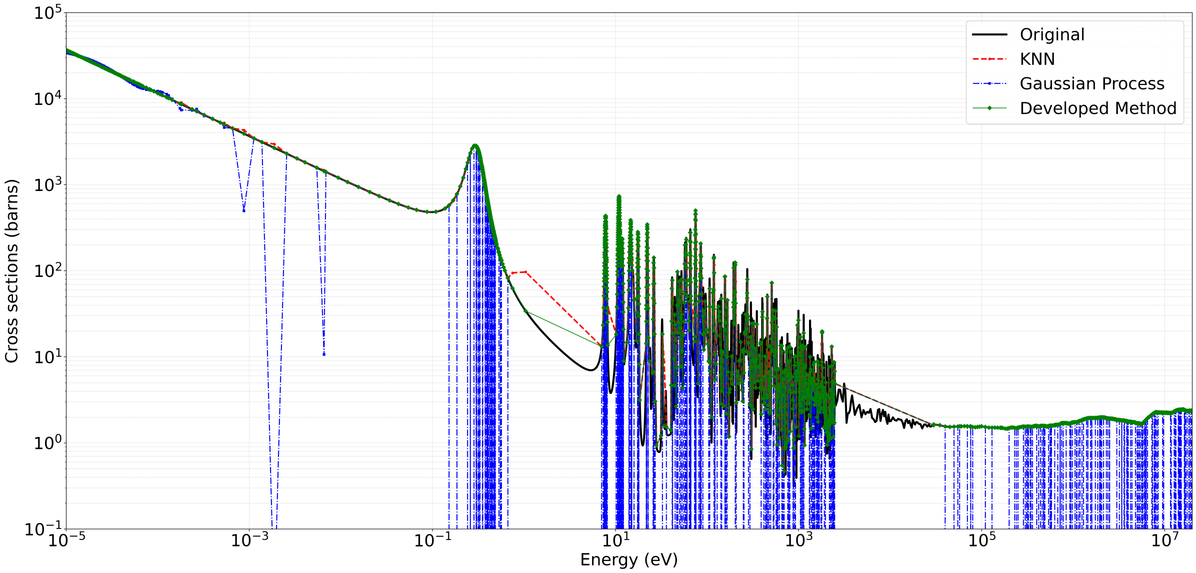

Figure 13. Comparison of Original, Developed Method, KNN, and GP for Pu-239 MT 18 (n, f) at 2500 K in ENDF/B-VII.1

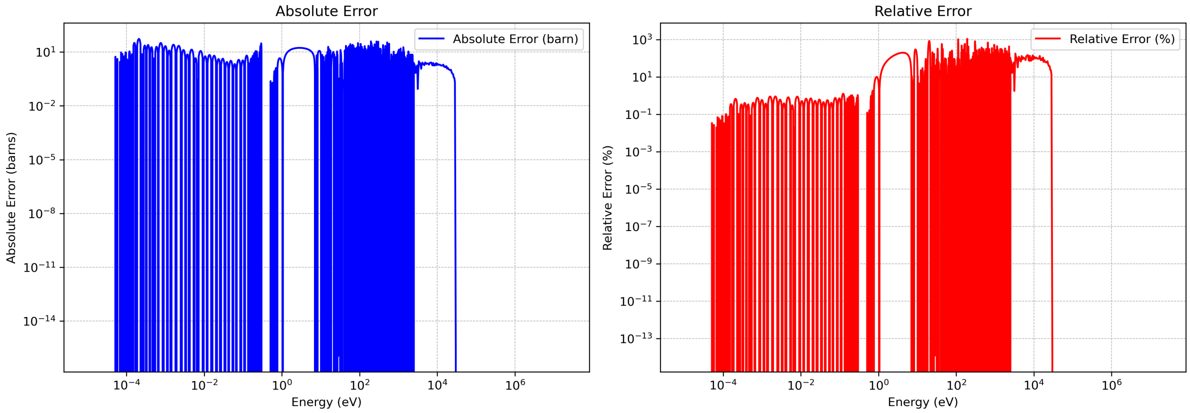

Figure 14. Absolute and Relative Errors of Developed Method for Pu-239 MT 18 (n, f) at 2500 K in ENDF/B-VII.1

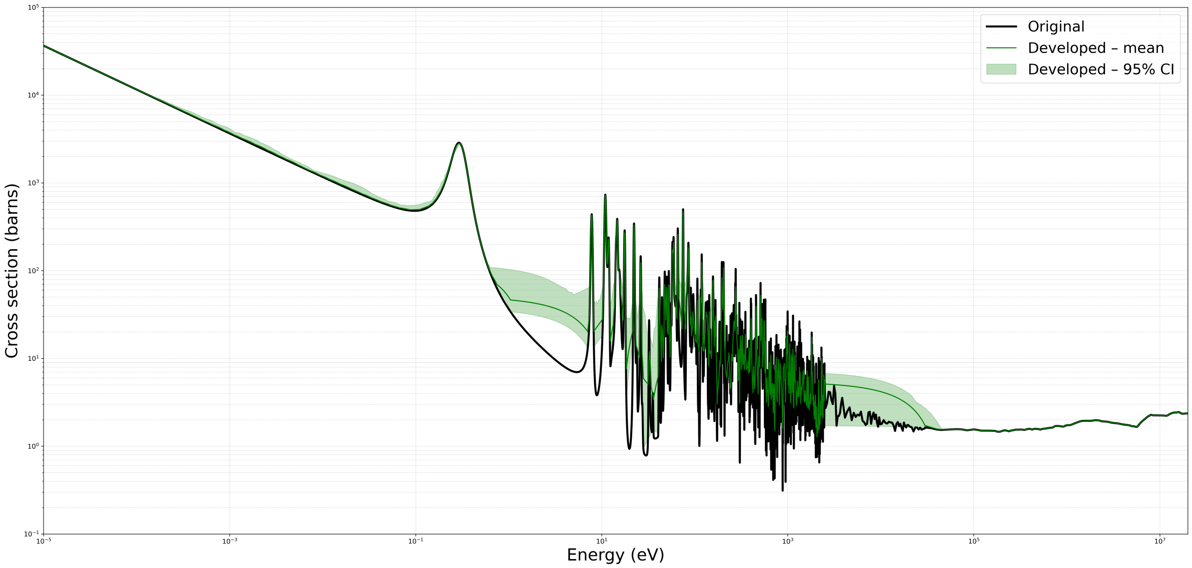

Figure 15. 95 % bootstrap confidence envelope for Pu-239 MT 18 (n, f) at 2500 K in ENDF/B-VII.1

Pu-239 from JEFF 3.3 Nuclear Data Library

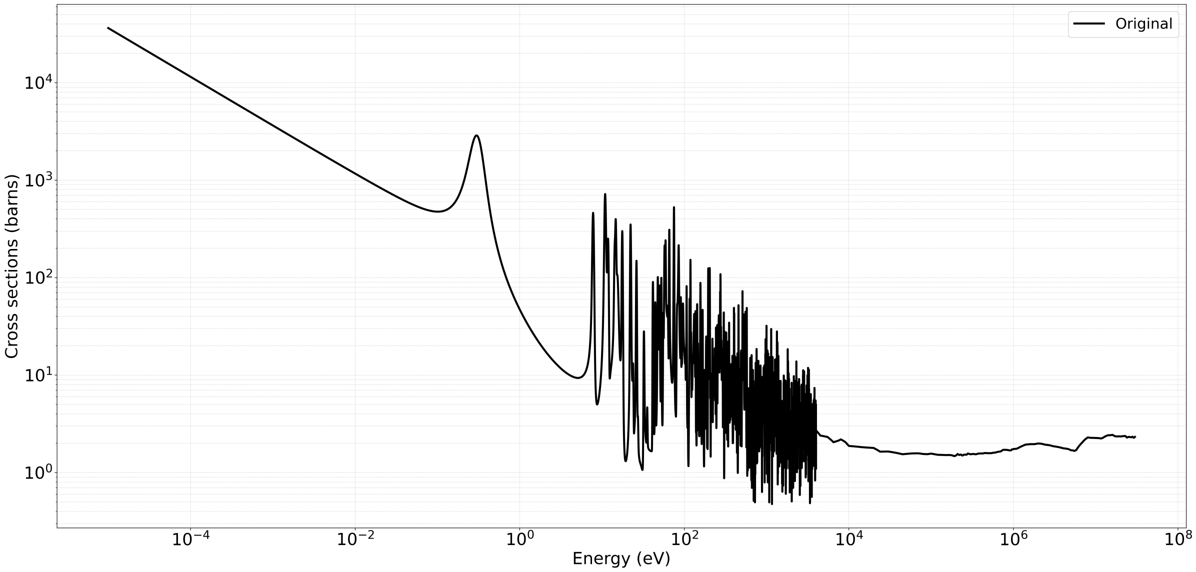

Figure 16. Original Data for Pu-239 MT 18 (n, f) at 2500 K in JEFF 3.3

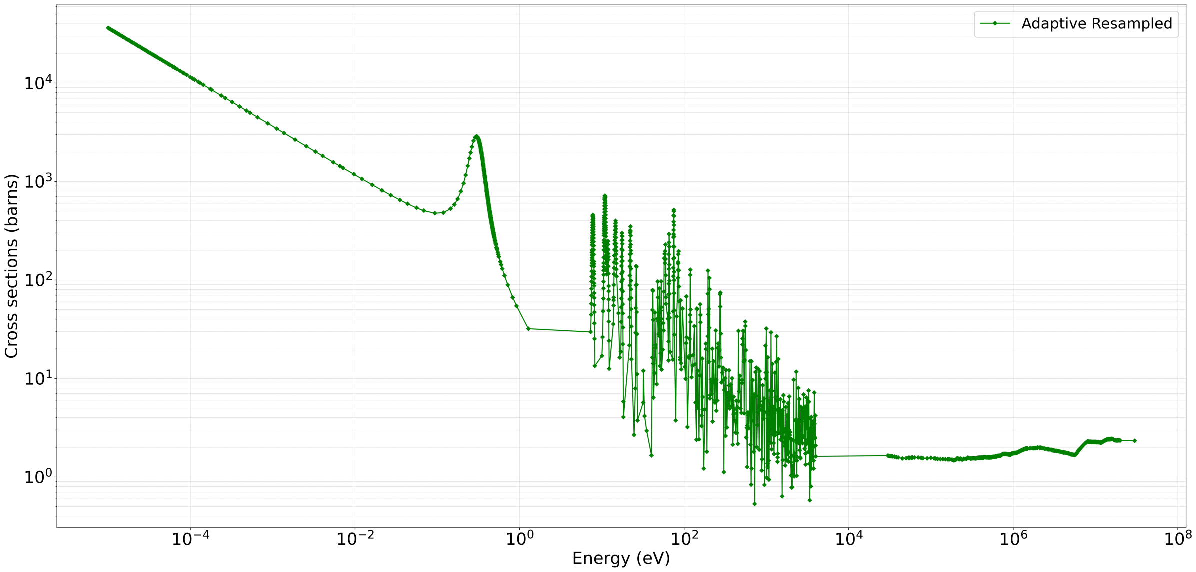

Figure 17. Resampled Developed Method for Pu-239 MT 18 (n, f) at 2500 K in JEFF 3.3

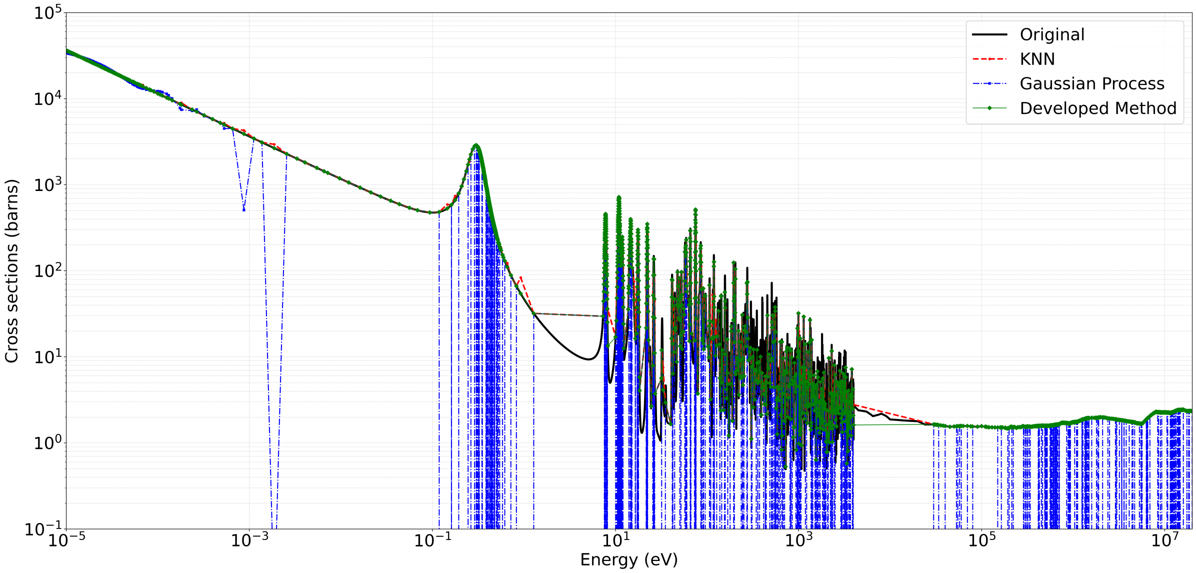

Figure 18. Comparison of Original, Developed Method, KNN, and GP for Pu-239 MT 18 (n, f) at 2500 K in JEFF 3.3

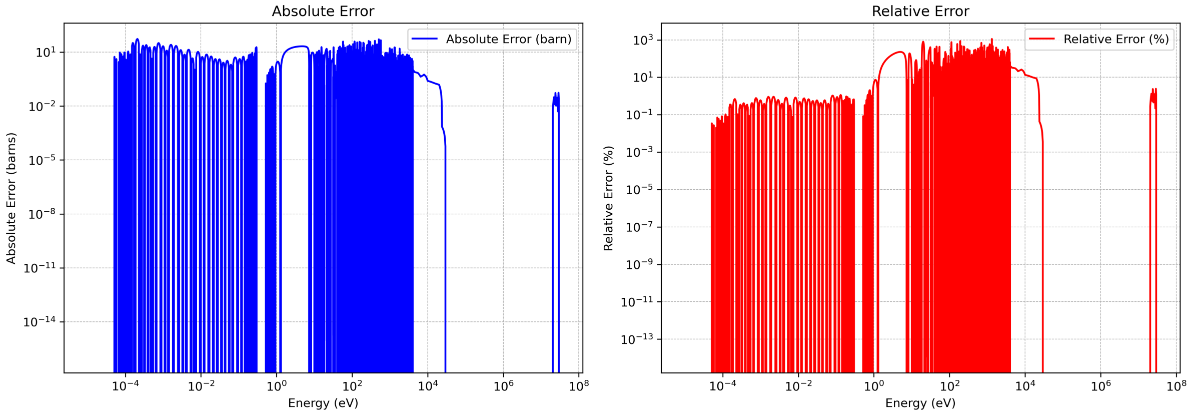

Figure 19. Absolute and Relative Errors of Developed Method for Pu-239 MT 18 (n, f) at 2500 K in JEFF 3.3

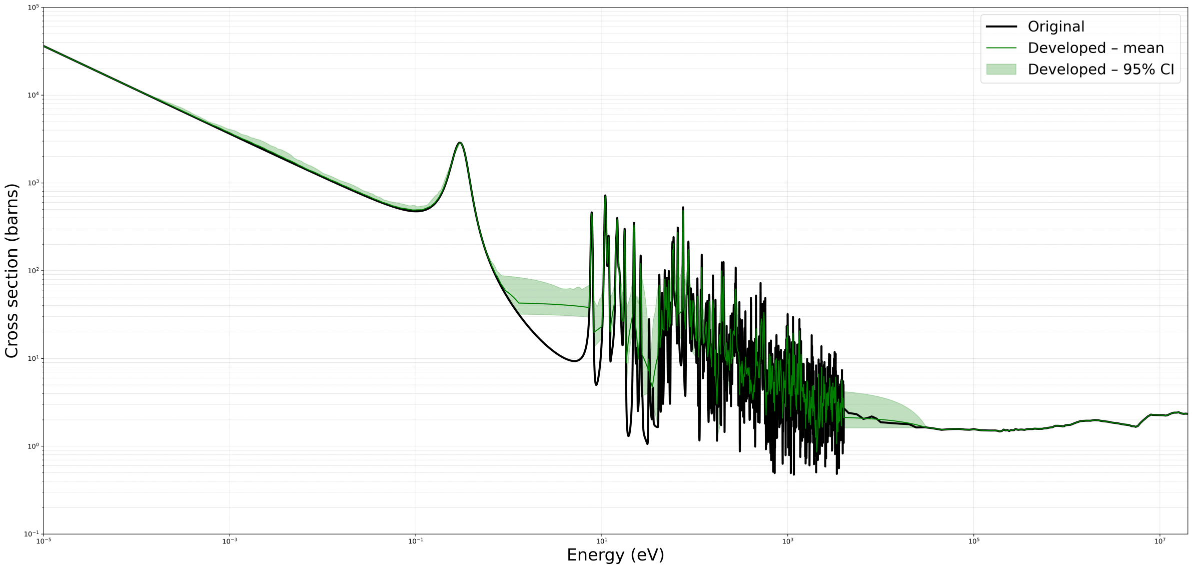

Figure 20. 95 % bootstrap confidence envelope for Pu-239 MT 18 (n, f) at 2500 K in JEFF 3.3

U-235 from JEFF 3.3 Nuclear Data Library

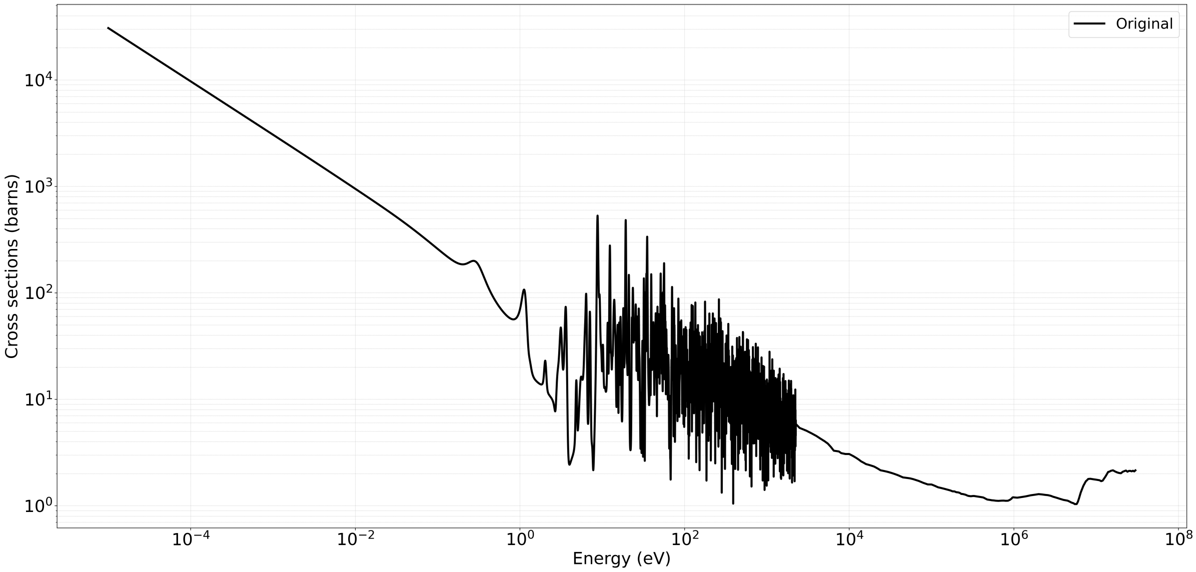

Figure 21. Original Data for U-235 MT 18 (n, f) at 1200 K in JEFF 3.3

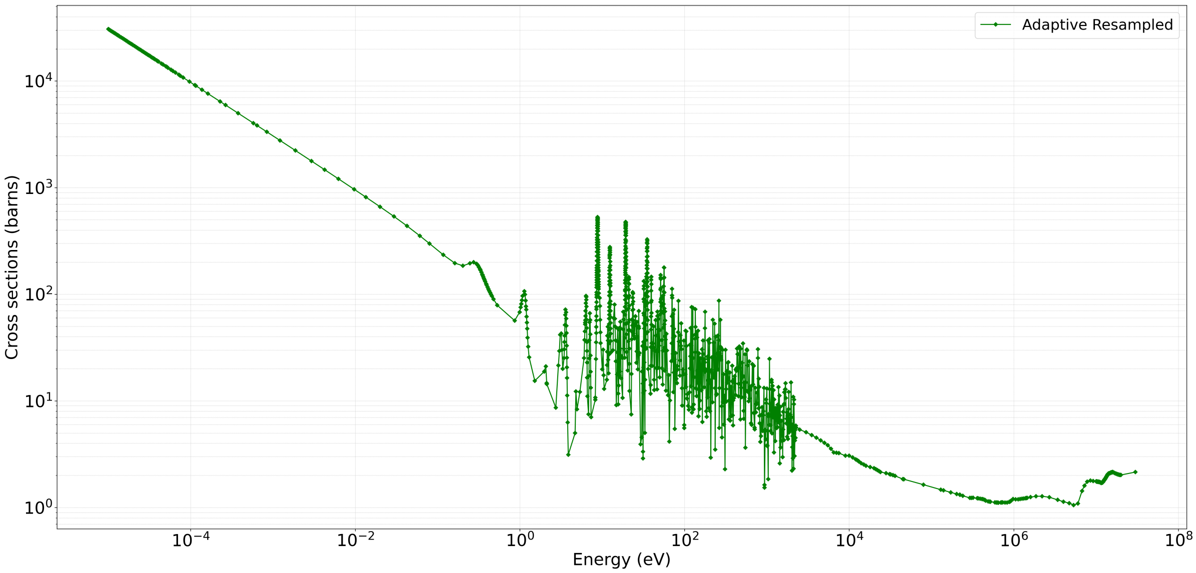

Figure 22. Resampled Developed Method for U-235 MT 18 (n, f) at 1200 K in JEFF 3.3

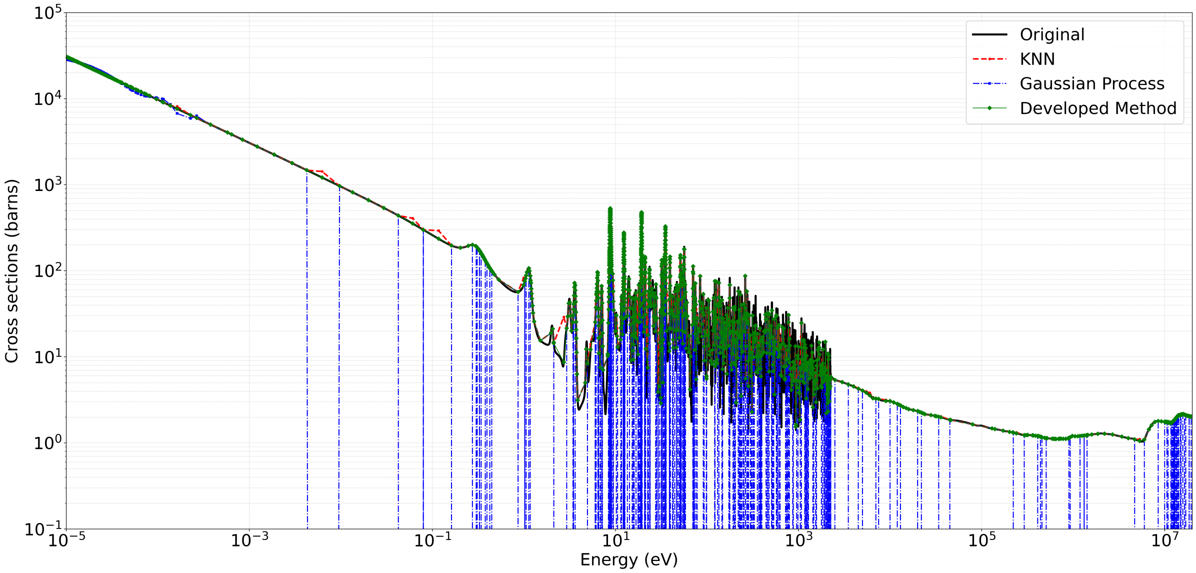

Figure 23. Comparison of Original, Developed Method, KNN, and GP for U-235 MT 18 (n, f) at 1200 K in JEFF 3.3

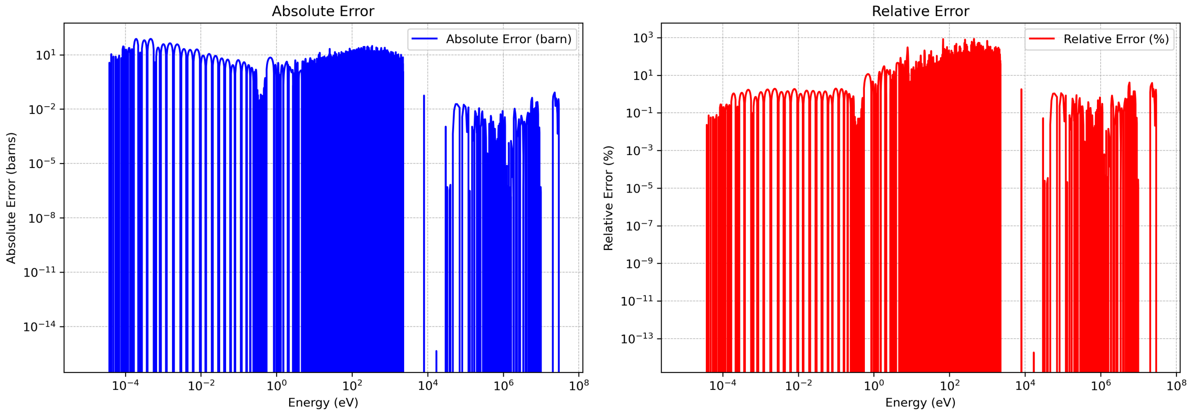

Figure 24. Absolute and Relative Errors of Developed Method for U-235 MT 18 (n, f) at 1200 K in JEFF 3.3

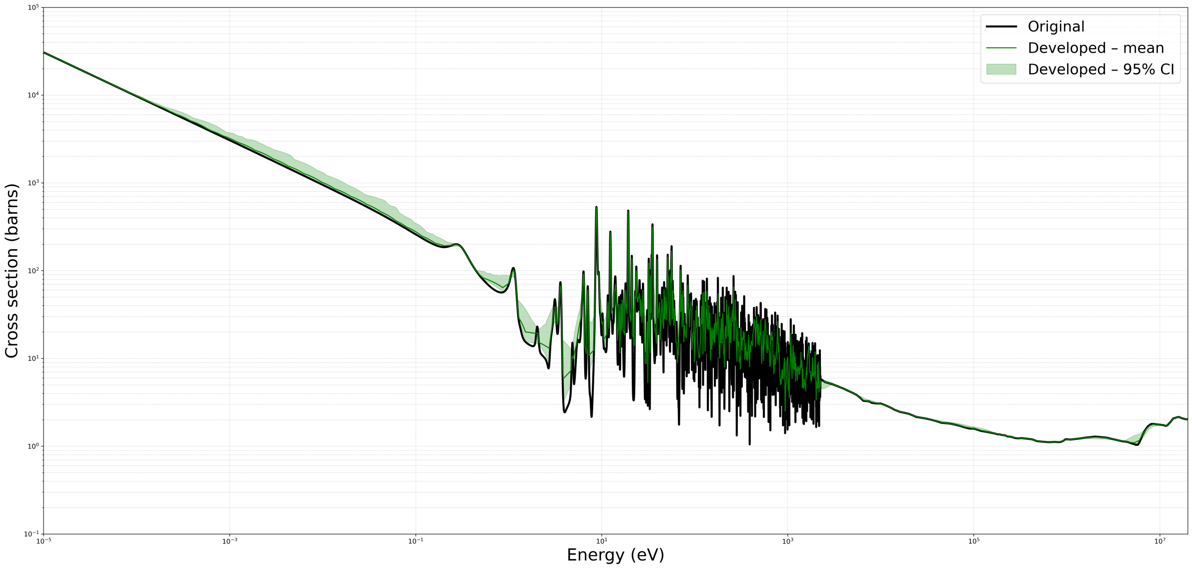

Figure 25. 95 % bootstrap confidence envelope for U-235 MT 18 (n, f) at 1200 K in JEFF 3.3

U-235 MT 2 from ENDF/B-VII.1 Nuclear Data Library

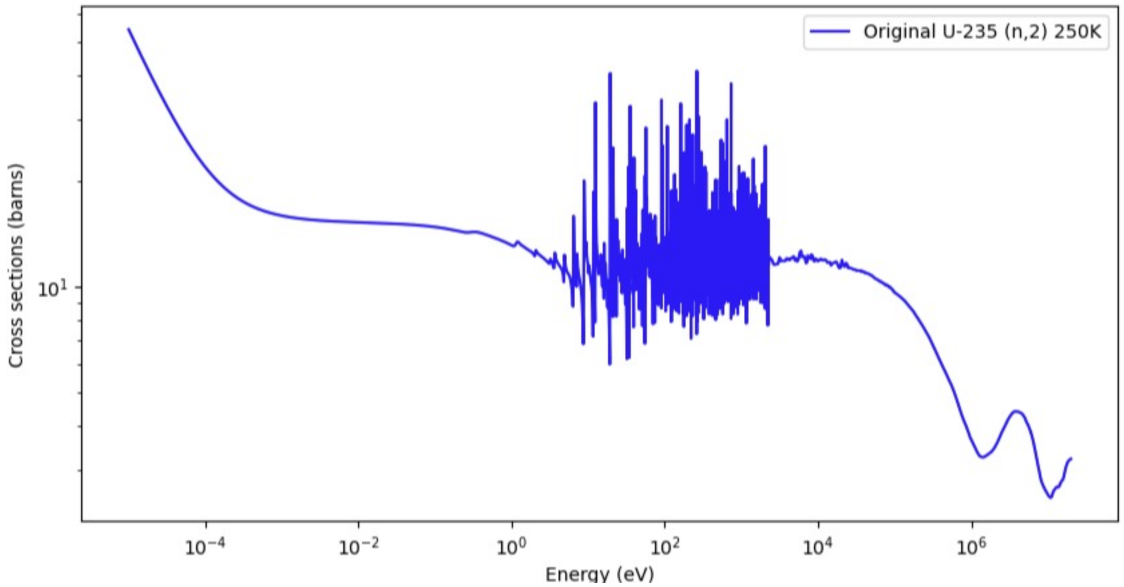

Figure 26. Original Data for U-235 $(n, 2)$ at 250 Kin ENDF/B-VII.1

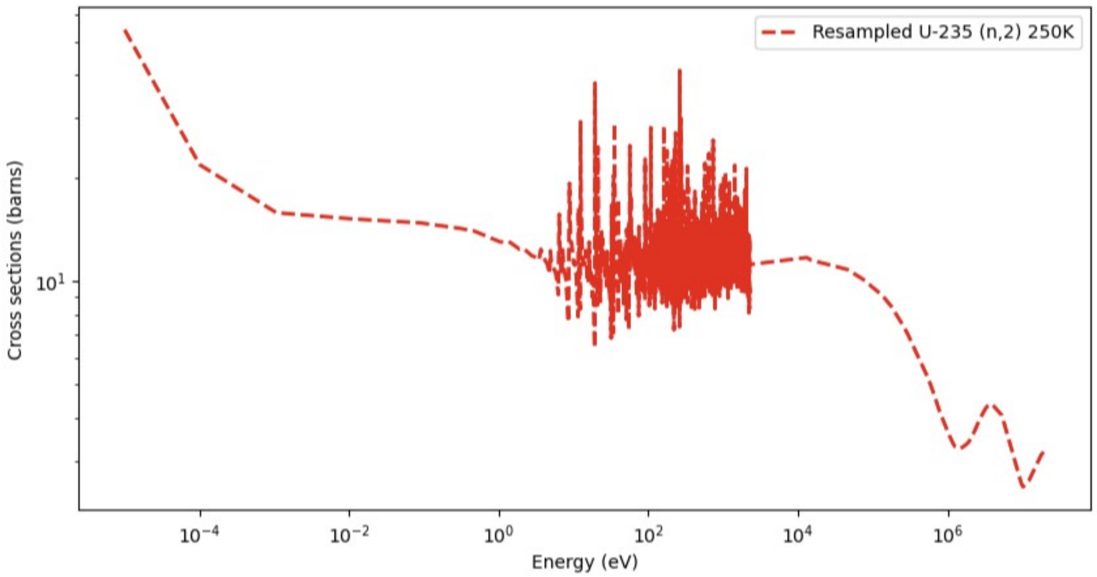

Figure 27. Resampled Developed Method for U-235 $(n, 2)$ at 250 K in ENDF/B-VII.1

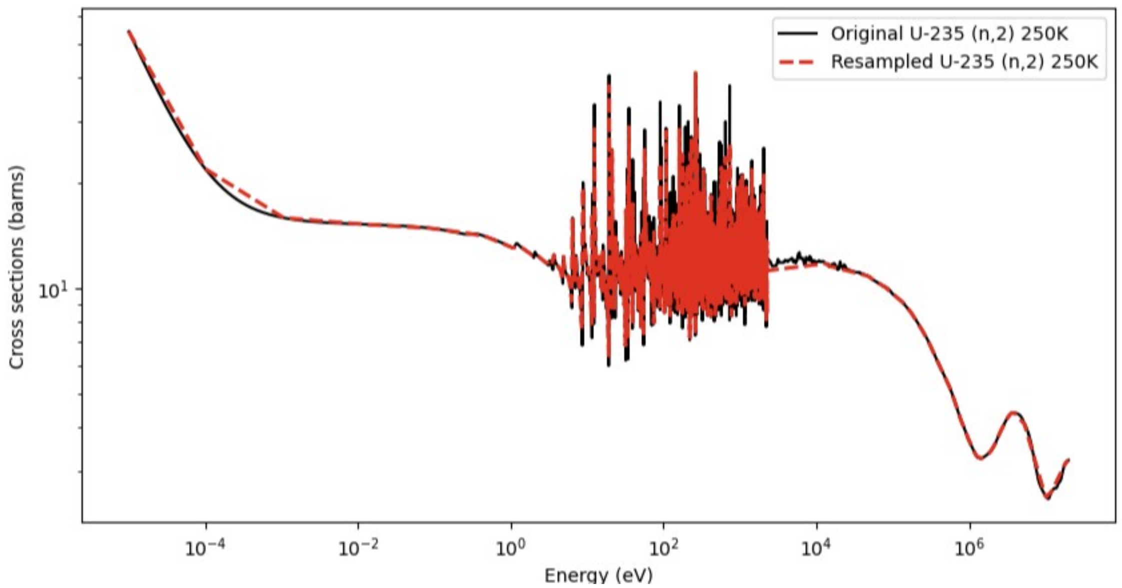

Figure 28. Comparison of Original and Developed Resampled Method for U-235 $(n,2)$ at 250 K in ENDF/B-VII.1

U-238 MT 102 from ENDF/B-VII.1 Nuclear Data Library

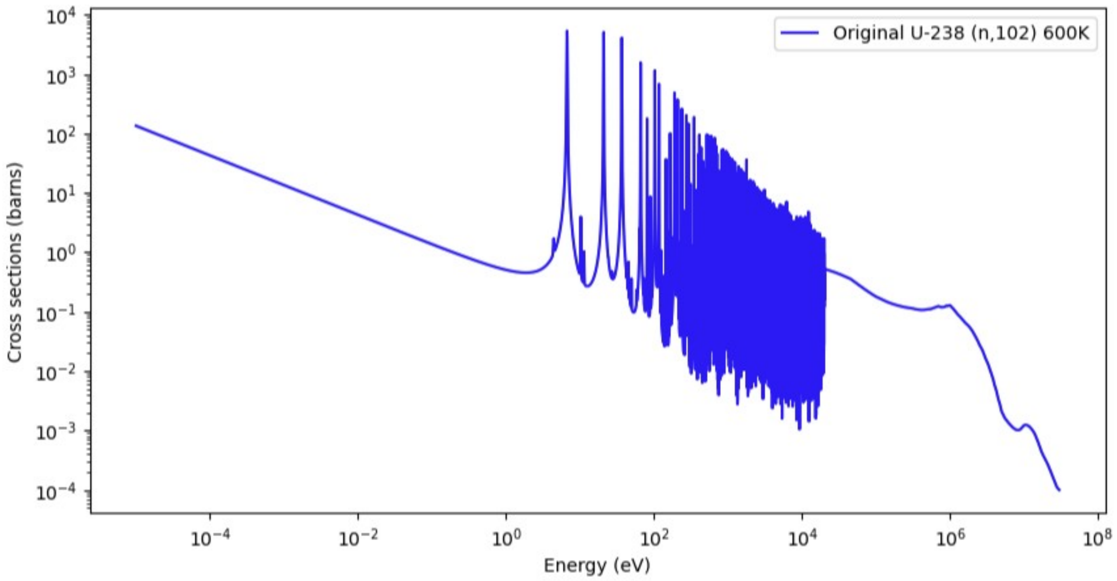

Figure 29. Original Data for U-238 $(n, 102)$ at 600 K in ENDF/B-VII.1

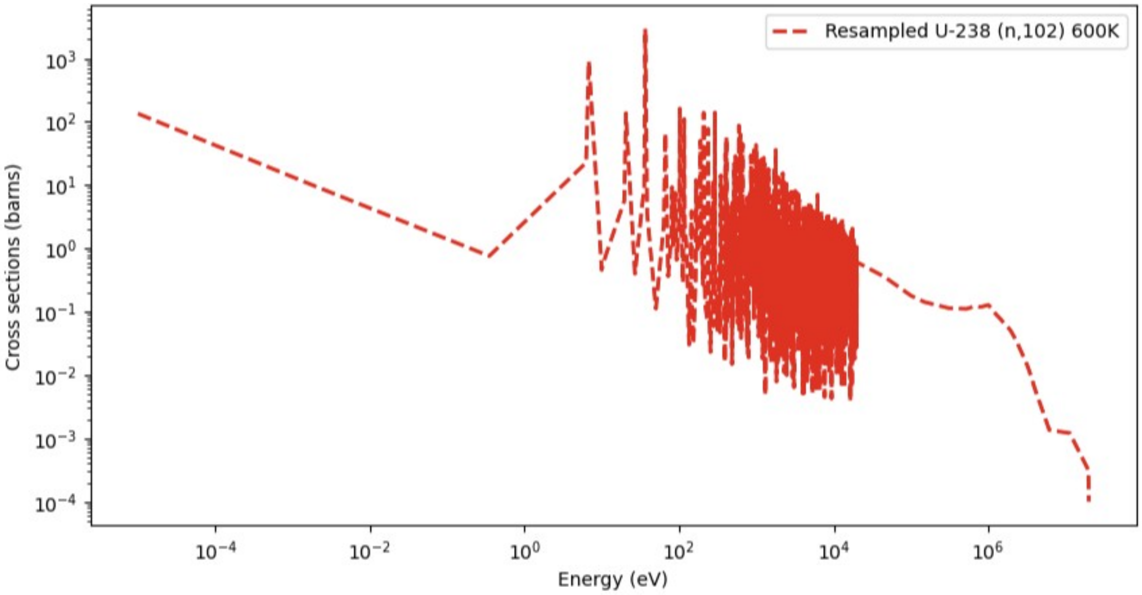

Figure 30. Resampled Developed Method for U-238 $(n, 102)$ at 600 K in ENDF/B-VII.1

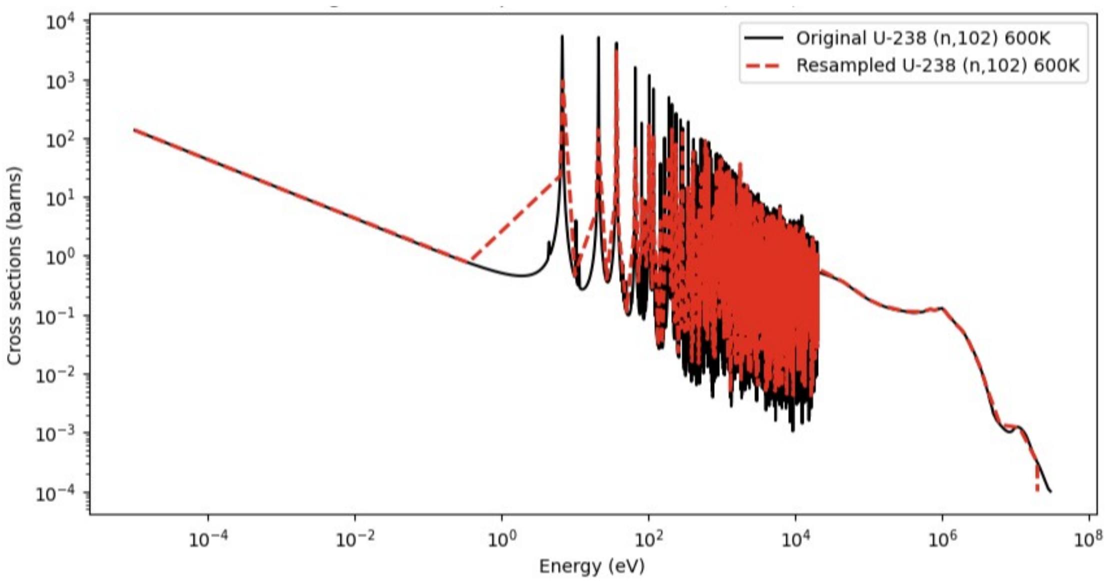

Figure 31. Comparison of Original and Developed Resampled Method for U-238 $(n, 102)$ at 600 K in ENDF/B-VII.1
